# Cancer-associated fibroblasts expressing FSTL3 promote vasculogenic mimicry formation and drive colon cancer malignancy

**DOI:** 10.1038/s41419-025-08009-w

**Published:** 2025-10-06

**Authors:** Leqian Ying, Yini Zhu, Lu Zhang, Min Ji, Meidan Wang, Lei Dong, Zhengcheng Yun, Yanping Chen, Jingyi Zhou, Chunchun Huang, Shengwei Zhang, Xuhong Yang, Hui Yang, Guichun Huang, Shukui Qin, Jinbing Xie, Lin Liu

**Affiliations:** 1 Department of Oncology, Zhongda Hospital, Medical School, Southeast University, Nanjing, 210009 Jiangsu China; 2 Department of Microbiology and Immunology, Medical School of Southeast University, Nanjing, 210009 Jiangsu China; 3 Department of Radiation Oncology, University of Freiburg Faculty of Medicine, Freiburg, 79106 Germany; 4 College of plant protection, Yangzhou university, Yangzhou, 225100 Jiangsu China; 5 Nurturing Center of Jiangsu Province for State Laboratory of AI Imaging & Interventional Radiology; Basic Medicine Research and Innovation Center of Ministry of Education; State Key Laboratory of Digital Medical Engineering; Department of Radiology, Zhongda Hospital, Medical School of Southeast University, Nanjing, 210009 Jiangsu China; 6 Department of Biochemistry and Molecular Biology, Medical School of Southeast University, Nanjing, 210009 Jiangsu China; 7 GI Cancer Center, Nanjing Tianyinshan Hospital, China Pharmaceutical University, Nanjing, 211100 Jiangsu China

**Keywords:** Extracellular signalling molecules, Colon cancer, Tumour biomarkers, Targeted therapies, Prognostic markers

## Abstract

Anti-angiogenic therapies are commonly employed in colon cancer management, yet many patients eventually develop resistance and experience disease progression. Vasculogenic mimicry (VM)—the formation of tumor-derived vessel-like networks—has been recognized as one mechanism contributing to this resistance, although the underlying details remain incompletely understood. Here, by integrating bioinformatic analyses of publicly available datasets and validating the results in patient samples (n = 157), we identified follistatin-like 3 (FSTL3) as a critical factor predominantly expressed in colon cancer-associated fibroblasts (CCAFs), with its expression strongly correlating with increased VM formation, intratumoral blood vessels, and poor prognosis. Single-cell RNA sequencing of tumors from VM and non-VM patients revealed that hypoxia drives FSTL3 expression in CCAFs, leading to extracellular matrix remodeling and enhancing cancer cell endothelial-like plasticity. Mechanistically, FSTL3 binds to transferrin receptor (TfR1), an iron-uptake receptor on cancer cells, thereby activating the TfR1/AKT/mTOR pathway and elevating VE-Cadherin to support endothelial-like transformation, VM, and metastatic progression. Notably, FSTL3-targeting antibodies (aFSTL3) effectively inhibited VM and angiogenesis in both in vitro and in vivo models, while the combination of aFSTL3 with bevacizumab produced synergistic suppression of neovascular-like structures and distant metastases. These findings demonstrate a pivotal role for FSTL3+ CCAFs in facilitating VM through TfR1-mediated signaling and offer a promising dual-target approach to overcome anti-angiogenic therapy resistance in colon cancer.

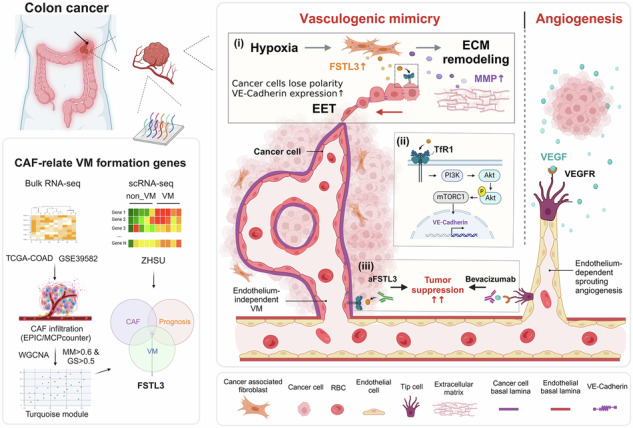

## Introduction

Colon cancer remains one of the most lethal malignancies globally, representing the leading cause of cancer-related deaths in men and the second leading cause in women under 50 years of age [1]. It is highly vascularized and heterogeneous, and despite advancements in anti-vascular endothelial growth factor (VEGF) therapies, resistance to these strategies—whether inherent or acquired—has significantly limited their efficacy [2–6]. Consequently, there is an urgent need to explore alternative, VEGF-independent mechanisms of tumor vascularization.

One such mechanism is vasculogenic mimicry (VM), a process in which tumor cells themselves form perfusable, vascular-like channels, thereby maintaining blood supply independently of traditional endothelial cell-mediated angiogenesis and VEGF signaling [7]. VM has been implicated in the limited efficacy of VEGF-targeted therapies and is observed in various aggressive malignancies, including melanoma, breast, ovarian, gastric, lung, and prostate cancers, where it is strongly associated with poor prognosis [7–13]. Unlike conventional endothelial-lined vessels, VM structures are formed exclusively by cancer cells through mechanisms such as epithelial-mesenchymal transition (EMT) [14], cancer stem cell (CSC) differentiation [15], and epithelial-endothelial transition (EET) [16]. These distinctive pathways underscore VM as a promising yet underexplored therapeutic target in cancer [17–22]. In colon cancer, VM is similarly linked to adverse clinical outcomes, although the molecular mechanisms driving its formation remain poorly understood [23–25].

The tumor microenvironment (TME) plays a critical role in cancer progression, with cancer-associated fibroblasts (CAFs) emerging as key regulators of this dynamic interplay [26–32]. Colon cancer-associated fibroblasts (CCAFs) exhibit significant heterogeneity, with distinct subtypes exerting either tumor-promoting or tumor-suppressing effects [30–33]. This functional diversity arises from the CAFs’ varied origins, dynamic plasticity, and complex interactions within the TME [34]. Notably, CAFs have been implicated in VM formation across multiple cancer types, including glioma, gastric, bladder, lung, and liver cancers [7, 8, 35–38]. Mechanistically, CAFs promote VM by secreting factors such as TGF-β, upregulating VE-Cadherin and matrix metalloproteinase-2 (MMP2), or inducing pro-VM genes like NOX4 [8, 37]. However, the role of CCAFs in VM formation in colon cancer remains largely unexplored. Elucidating the molecular drivers linking CCAFs to VM could unveil novel therapeutic opportunities.

Follistatin-like 3 (FSTL3), a secreted glycoprotein belonging to the follistatin-module-protein family, is recognized as an oncogene. It activates EMT via BMP/SMAD signaling in gastric cancer [39, 40] and antagonizes endogenous activin effects to promote breast tumor cell proliferation [41]. FSTL3 is known to bind and antagonize members of the TGF-β family, including activin, bone morphogenetic protein 2, and myostatin [41, 42]. In colon cancer, FSTL3 is upregulated and promotes tumor progression by activating the TGF-β pathway [43–45]. However, most studies have focused on its tumor-intrinsic roles, leaving its function in the tumor stroma and VM largely unexplored.

In this study, we identify FSTL3 as a key regulator of VM in colon cancer, predominantly expressed by CCAFs. We demonstrate that hypoxia significantly upregulates FSTL3 expression, driving extracellular matrix (ECM) remodeling and promoting cancer cell EET transformation. This highlights an alternative tumor response to hypoxia beyond VEGF-driven angiogenesis. Mechanistically, CCAF-secreted FSTL3 binds to the transferrin receptor (TfR1) on tumor cells, mediating CCAF-driven VM through the FSTL3/TfR1 signaling axis. These findings provide novel insights into the molecular mechanisms of VM formation and propose FSTL3 as a potential therapeutic target to combat colon cancer progression.

## Materials and methods

### Clinical samples and data collection

Bulk RNA-sequencing (RNA-seq) data were obtained from the Cancer Genome Atlas (TCGA) and the Gene Expression Omnibus Database (GEO, RRID: SCR_005012). Specifically, datasets from TCGA-COAD (*n* = 446), GSE39582 (*n* = 562), and GSE17536 (*n* = 177) were downloaded, including comprehensive clinical data. Fragments per kilobase per million (FPKM) values were converted to transcripts per million (TPM) for normalization. Batch effects were corrected using the “ComBat” function from the “sva” R package (v3.50.0) [46] after log2 transformation of TPM values (log_2_[TPM + 1]). Inclusion criteria for the datasets were as follows: (1) Availability of clinical information, including overall survival (OS), age, gender, and tumor stage; (2) Confirmed pathological diagnosis of colon cancer; (3) No prior history of radiation therapy, chemotherapy, targeted therapy, immunotherapy, or other anticancer treatments (including neoadjuvant therapy).

Formalin-fixed, paraffin-embedded (FFPE) tissue blocks were retrospectively collected from 157 colon cancer patients who underwent radical surgery at Zhongda Hospital, Affiliated to Southeast University (ZHSU), between January 1, 2014, and August 31, 2021. Follow-up data were complete, with the last follow-up date recorded as September 1, 2023. Additionally, fresh tumor tissues and matched normal colon tissues were obtained from 4 colon cancer patients. Inclusion criteria for tissue samples: (1) Pathologically confirmed diagnosis of colon cancer; (2) Availability of fully preserved FFPE tissue blocks; (3) No history of anticancer therapy prior to sample collection. Exclusion criteria: (1) Patients with severe comorbidities, including cardiopulmonary insufficiency, severe infections, hematologic disorders, autoimmune diseases, hepatitis, or other malignancies; (2) Cases lacking essential clinical or follow-up data.

All diagnoses were independently verified by two pathologists. Clinical staging was performed according to the American Joint Committee on Cancer (AJCC) Staging Manual, 8th edition. Detailed clinical characteristics of the cohort are provided in Table S1. This study was conducted in compliance with the Declaration of Helsinki. Written informed consent was obtained from all participants, and the study protocol was approved by the Clinical Research Ethics Committee of ZHSU.

### Tissue dissociation and single-cell RNA sequencing (scRNA-seq)

Gene expression matrices were obtained from both public and local cohorts, respectively. Tumor Immune Single-cell Hub 2 (TISCH2) was used to explore the expression levels of FSTL3 and SERPINE1 in different cell types of EMTAB8107, GSE146771, GSE166555 and GSE179784 cohorts (http://tisch.comp-genomics.org/). The public data (*n* = 20) analyzed in this study were obtained from GSE236581 cohorts [47].

Fresh colon cancer tissues were procured from patients and subjected to a wash with ice-cold phosphate-buffered saline (PBS). Subsequently, these tissues were processed using a tumor dissociation kit (Miltenyi Biotec, Germany) to generate single-cell suspensions. Prior to the preparation of single-cell suspensions, the tissues obtained from surgical resection were bisected. One portion was utilized for the preparation of frozen sections to evaluate VM via CD31/PAS staining, while the other portion was used to prepare single-cell suspensions for conducting scRNA-seq. The local cohort, included tumor tissue specimens from VM (*n* = 1) and non-VM (*n* = 1) patients, was processed with the DNBelab C4 system (BGI, Shenzhen, China). The construction of cDNA libraries, library purification, and transcriptome sequencing were conducted following the protocols provided by the Majorbio Company (Shanghai, China) [48].

The “Seurat” R package (v4.3.0) was primarily utilized to analyze the scRNA-seq data based on a standardized procedure [49]. Genes expressed in fewer than three cells, cells with fewer than fifty detected genes, and cells with mitochondrial percentages exceeding 5% were excluded from the analysis. The count matrix was normalized with default settings, and top 1500 variable genes from each dataset were scaled for principal components analysis (PCA). The Harmony algorithm was used to combine all samples and reduce batch effects, followed by principal component analysis on the integrated expression matrix for dimensional reduction. The first 20 principal components were computed, and cell clusters were identified at a resolution of 0.5. To annotate cell types based on marker genes, the “SingleR” R package (v1.0.0) was employed [50]. The “copyKAT” R package (v1.1.0) was employed to objectively distinguish epithelial cells as either normal epithelial cells or cancer cells [51]. The guidelines provided in the GitHub repository (https://github.com/navinlabcode/copykat) were followed, and “copyKAT” was used with default parameters. The results of the “copyKAT” tool analysis were presented in Table S2.

CCAFs subtypes-related markers referred to published literature [52] and the marker genes of “Matrix CAFs”, “Inflammatory CAFs”, “Vascular CAFs”, “Tumor-like CAFs”, “Antigen-presenting CAFs”, “Reticular-like CAFs” and “Dividing CAFs” respectively were MMP11, PLA2G2A, MCAM, PDPN, CD74, CCL21, and MKI67.

For cellular level validation, TPM-normalized RNA-Seq transcriptomic data of the genes in colorectal cancer cells (*n* = 56) and CCAFs (*n* = 2) were downloaded from the Cancer Cell Line Encyclopedia (CCLE) database [53].

### AUCell scores of hallmark gene sets

Gene set enrichment analysis (GSEA, RRID: SCR_003199) of hallmark gene sets (*n* = 65) was downloaded from the MSigDB database (*n* = 50) [54] and CancerSEA database (*n* = 14). Furthermore, a synthesis of key gene sets associated with EET is provided, drawing upon existing literature reports [16, 55]. The “AUCell” R package (v1.24.0) was used to calculate per-cell AUCell scores via the “aucell” function. AUCell ranked genes by their expression levels in each cell and assessed pathway activity based on the ranks of pathway-specific genes.

### Estimation of fibroblast infiltration

To evaluate the infiltration of fibroblasts in the TME, several computational methods were employed, including the Estimate of Proportions of Immune and Cancer Cells (EPIC) [56], Microenvironment Cell Populations-counter (MCP-counter) [57], xCell [58] and ESTIMATE [59]. These methods were implemented using the respective “EPIC”, “MCPcounter”, “xCell”, and “estimate” R packages, following protocols established in the literature. Additionally, based on established transcriptomic biomarkers, the Tumor Immune Dysfunction and Exclusion (TIDE) tool [60, 61] was utilized to assess CAFs infiltration via Pearson correlation analysis between expression profile and FAP^+^ CAF signature. The results pertaining to CCAF infiltration and Stromalscore were extracted from these five infiltration methods based on the mRNA expression profile of TCGA-COAD and GSE39582 cohorts. The results of five infiltration methods are presented in Table S3–4.

To investigate the abundance of FSTL3^+^ CCAFs from RNA-seq in TCGA_COAD and GSE39582 cohorts, the “MCP-counter” R package was used as previously described. Briefly, FSTL3^+^ CCAF signature genes were defined as FSTL3, ACTA2, and FAP. The infiltration scores of FSTL3^+^ CCAFs were then calculated, and their association with patient survival was evaluated.

### Weighted Gene Co-expression Network Analysis (WGCNA)

CCAFs hub genes that were strongly associated with CCAFs infiltration were mainly filtered and detected via “WGCNA” R package (v1.72-5, RRID: SCR_003302) [62]. First, Pearson’s correlation coefficients represented the direct correlation between gene pairs. The soft-thresholding power (β) was determined based on the scale-free topology criterion. We clustered the adjacency matrix using topological overlap to explore both direct and indirect correlations among module genes, and visualized the results using TOMplot, a tool that displayed the topological overlap matrix (TOM). This plot represented gene similarity based on co-expression patterns, with higher overlap values indicating stronger relationships between genes within a network module. Employing epigenetic significance (ES), we pinpointed the hub gene module most correlated with CAF infiltration and Stromal score by the “corPvalueStudent” function. With an optimal β value of 3 for TCGA-COAD and 4 for GSE39582, we constructed a hierarchical clustering tree, merging closely related modules according to the height value < 0.2. Finally, we defined hub genes as those with module membership (MM) > 0.6 and gene significance (GS) > 0.5, resulting in a final selection of 244 overlapping hub genes from both cohorts.

### Differential expression genes between VM and non-VM groups

Non-neuroendocrine cells have been shown to possess the ability to form VM structures compared to neuroendocrine cells. The VM-related genes were obtained from GSE240789 cohort based on comparing VM-competent (non-neuroendocrine) cells with VM-deficient (neuroendocrine) cells [63]. Differential gene expression (DGE) analysis was performed using DESeq2 (v1.18.1, RRID: SCR_000154), and genes with an adjusted *p* value < 0.01 and a Log_2_ fold change (Log_2_FC) > 2 cut-offs were considered significant VM-related differentially expressed genes.

### Functional enrichment and gene set enrichment analysis

The reference files required by gene set enrichment analysis (GSEA) [64] were downloaded from MsigDB. Enrichment analyses of KEGG (RRID: SCR_012773) pathways (c2.cp.kegg.v7.3.symbols) were performed on identifying the pathways through “clusterProfiler” R package (v3.14.3) [65]. Biological functions and pathways with an adjusted *p* < 0.05 were deemed to be significantly enriched.

### Cellchat analysis

The possible interactions among the cell populations based on the ligand-receptor pair data in CellChatDB were evaluated by “CellChat” R package (v2.1.2) [66].

### Cell lines and cell culture

Human colon cancer cell lines, HT-29 (RRID: CVCL_0320), RKO (RRID: CVCL_0504), and DLD-1 (RRID: CVCL_0248), were purchased from the Type Culture Collection of the Chinese Academy of Science (Shanghai, China), and HCT116 cells (RRID: CVCL_0291) were obtained from the Cell Resource Center, Peking Union Medical College (Beijing, China). The human colon epithelial cell line NCM460 (RRID: CVCL_0460) were obtained from the Department of Oncology of Jiangsu Cancer Hospital. The human colon fibroblast cell line CCD-18Co (RRID: CVCL_2379) and human umbilical vein endothelial cells (HUVEC, RRID: CVCL_2959) were purchased from the American Type Culture Collection (ATCC, Manassas, VA, USA). HCT116 cells were cultured in IMDM supplemented with 10% FBS, whereas HT-29 cells were cultivated in McCoy’s 5A medium supplemented with 10% FBS. RKO, DLD-1, NCM460, and HUVEC cell lines were cultivated in DMEM with 10% FBS. CCD-18Co cells were cultured in MEM/EBSS with 10% FBS. All culture media were supplemented with 1% penicillin/streptomycin, and cells were cultured in a humidified incubator at 37 °C and 5% CO2. All cell lines were tested negative for mycoplasma contamination.

### Primary fibroblast isolation and culture

Primary fibroblast cell lines from colon cancer (PCAF) and adjacent normal tissue (PNF) were cultured from two patients with colon cancer at the ZHSU, under a protocol approved by the Clinical Research Ethics Committee of the ZHSU. Approximately 2-cm^3^ pieces of tissue were minced and digested with collagenase IV (1 mg/mL, Gibco, Massachusetts, USA) and hyaluronidase (1 mg/mL, Sigma-Aldrich, St Louis, USA) for 1 h at 37 °C. The solution was centrifuged at 1000 rpm for 5 minutes and washed twice with PBS. The supernatant was discarded, and the remaining cells were resuspended and cultured in Dulbecco’s modified Eagle’s medium (Invitrogen Life Technologies, Carlsbad, USA) supplemented with 10% fetal bovine serum (Sigma-Aldrich), 1% penicillin, 1% streptomycin, and an anti-mycotic agent (Invitrogen Life Technologies). Primary cultures were passaged at least 3 times to remove colon cell contamination. Primary fibroblasts with passage numbers below 11 were used for in vivo and in vitro experiments.

### In vitro hypoxia model

A cell hypoxia model was established using a modular incubator chamber. Briefly, PCAF was placed in the hypoxic chamber with a 1% O₂ gas mixture, while PCAF in normoxia was used as a control and incubated at 37 °C for 24 h.

### CM collection

PCAF, PNF, and other colon cell lines were seeded in 6-well plates (2.0 × 10^5^ cells/well) and incubated overnight. When 70–80% confluence was reached, the cells were rinsed twice with serum-free DMEM and cultured in this medium for 48 h. Then, the supernatant was centrifuged at 4000 rpm at 4 °C for 10 min and filtered through a 0.22 μm filter to remove cellular debris.

### Enzyme-linked immunosorbent assay (ELISA)

Clinical plasma specimens from colon cancer patients (*n* = 30) and colon adenoma donors (*n* = 10) were collected from the biobank of the ZHSU throughout the same period, and signed informed consent was obtained from all patients. Plasma samples were stored at −80 °C before use. ELISA was performed using a sandwich enzyme immunoassay method with a pre-coated 96-well strip plate from an ELISA kit for FSTL3 (Proteintech, #KE00258, Wuhan, China), according to the manufacturer’s instructions. Plasma specimens and the CM of cell lines were diluted 20-folds before analysis.

### RNA extraction and RT-quantitative PCR (RT-qPCR)

TRIzol reagent (Invitrogen Life Technologies) was used to extract total RNA from cell lines. cDNA was synthesized using reverse transcription (Vazyme, Nanjing, China) according to the manufacturer’s instructions. PCR was performed in duplicate on the RT product using SYBR Green qPCR Master Mix (Vazyme) with the primer pairs in a total reaction volume of 20 μL. The reactions were analyzed using an Applied Biosystems StepOnePlus System. Dissociation curves were run for all reactions to ensure the amplification of a single product with the appropriate melting temperature. FC was computed relative to the control using the 2^−ΔΔCT^ method [67]. The primer sequences are listed in Table S5.

### Protein extraction and Western blotting

Proteins from fresh tissues and cell lines were treated with RIPA buffer containing protease and phosphorylase inhibitors. The protein concentration was determined by the BCA method. According to standard protocols, western blot analysis was conducted using anti-FSTL3 antibody (Abcepta, #AP12300b, 1:2000, Suzhou, China, RRID: AB_10821490), anti-AKT (Wanleibio, #WL0003b, 1:1000, Beijing, China, RRID: AB_2833233), anti-pAKT (Ser473) (Cell signaling technology, #4060 T, 1:2000, Boston, USA, RRID: AB_2315049), anti-VE-Cadherin (Wanleibio, #WL02033, 1:1000, RRID: AB_3076321), anti-mTOR (Proteintech, #66888-1-Ig, 1:5000, RRID: AB_2882219), anti-p-mTOR (Proteintech, #67778-1-Ig, 1:2000, RRID: AB_2889842), anti-S6 (Proteintech, #80208-1-RR, 1:5000, RRID: AB_2918876), anti-p-S6 (Proteintech, #67898-1-Ig, 1:5000, RRID: AB_2918654) and GAPDH (Abcam, #ab181602, 1:10000, Cambridge, UK, RRID: AB_2630358). In brief, SDS-PAGE gels were used to separate proteins, which were then transferred onto PVDF membranes (Millipore, Bedford, MA, USA). Subsequently, the membranes were blocked with 5% non-fat dry milk and incubated with the primary antibody overnight at 4 °C. The secondary antibodies corresponded to each protein primary antibody, and immunoblots were probed with an ECL detection reagent.

### RNA quality control, library preparation, and sequencing

HCT116 cells were divided into control (*n* = 3) and FSTL3 treatment (*n* = 3) groups. RNA was extracted and assessed for purity and integrity using 1% agarose gel electrophoresis, NanoPhotometer® spectrophotometer (IMPLEN, CA, USA), and the RNA Nano 6000 Assay Kit on the Bioanalyzer 2100 (Agilent Technologies, CA, USA). Library preparation was performed with 1 µg of RNA using the NEBNext® Ultra™ RNA Library Prep Kit for Illumina® (NEB, USA). mRNA was purified using poly-T oligo-attached beads, fragmented, and reverse transcribed to cDNA. The cDNA was size-selected (250–300 bp), and libraries were assessed for quality before sequencing on the Illumina Novaseq platform with 150 bp paired-end reads. Sequencing was performed using the Sequencing by Synthesis (SBS) method, where dNTPs, DNA polymerase, and adapter primers were used to amplify the flow cell. Fluorescent signals were captured to obtain the RNA-Seq data.

### RNA-seq data analysis of HCT116 cells

Raw reads were processed to remove adapters, poly-N sequences, and low-quality reads (defined as reads where > 50% of bases have a Qphred ≤ 20), generating clean data. Reads were aligned to the reference genome using HISAT2 (v2.0.5, RRID: SCR_015530). Gene expression was quantified with featureCounts (RRID: SCR_012919), and FPKM values were calculated. Differential expression analysis was performed using DESeq2 (v1.16.1), with significance thresholds set at adjusted *p* < 0.05 (Benjamini-Hochberg method) and |Log_2_FC| > 0. KEGG pathway enrichment analysis of differentially expressed genes (*n* = 240) was conducted using the “clusterProfiler” R package as described above. Proteins associated with the HIF-1 signaling pathway (hsa04066) were retrieved from the KEGG database.

### Coimmunoprecipitation (Co-IP) and Silver Staining

Co-IP of FSTL3-associated proteins was performed using the Pierce^TM^ Classic Magnetic IP/Co-IP Kit (Thermo Fischer Scientific, #88804, MA, USA) according to the manufacturer’s instructions. HCT116 cells were cultured in 10 cm plates, and 1 mL of IP Lysis buffer (50 mM Tris-HCl pH 7.4, 1 mM EDTA, 50 mM NaCl, 1% Triton X-100) containing Thermo Scientific Halt Protease Inhibitor Cocktail (Thermo Fisher Scientific) was added. The plates were incubated with the primary antibody overnight at 4 °C. The next day, the antibody-bound protein of interest in lysis buffer was incubated with MagnaBind Protein A/G Beads (Thermo Fisher Scientific). After three washes with Wash Buffer (0.5 M Tris-HCl pH 7.4, 1.5 M NaCl), protein-bound beads were mixed with loading buffer (Fdbio science) to the final concentration of loading buffer and boiled for 10 minutes at 95 °C. The samples were then stored at −20 °C or ready for SDS-PAGE (sodium dodecyl sulfate-polyacrylamide gel electrophoresis).

For silver staining, equal amounts of the protein complex above were loaded on 15% SDS-PAGE. Then the gel was stained using the Rapid silver staining kit (Beyotime, Shanghai, China) according to the manufacturer’s instructions. The differential band indicated by silver staining was excised from the CO-IP of FSTL3 and IgG groups, and sent for mass spectrometric analyses.

### Mass spectrometric analyses

The pulled-down proteins were analyzed by liquid chromatography-tandem mass spectrometry (LC-MS/MS) in FitGene Biotechnology Co., Ltd. (Guangzhou, China). The peptides were subjected to nanoelectrospray ionization followed by MS/MS analysis using a Q Exactive mass spectrometer (Thermo Fisher Scientific) coupled online to the liquid chromatography. Protein identification was performed with MASCOT (RRID: SCR_014322) by searching the Uniprot database (RRID: SCR_002380). Peptide mass tolerance values were set to 20 ppm and fragment mass tolerances were of 0.6 Da. The potential proteins (*n* = 732) were selected by excluding the proteins pulled down in the IgG control group from the FSTL3 group. Additionally, a list of human plasma membrane proteins (*n* = 1347) was obtained from the Membranome database [68, 69].

### Cell transfection

For small hairpin RNA (shRNA)-mediated FSTL3 knockdown (shFSTL3), the target RNAi sequence, 5’-GCCTTCCCTGCAAAGATTCGT-3’ for PCAF, was synthesized and cloned into the expression vector pGCSIL-green fluorescent protein (GFP) by GeneChem Company (Shanghai, China). The non-target RNAi sequence, 5’-TTCTCCGAACGTGTCACGT-3’, was employed to generate the negative control. In compliance with the manufacturer’s recommended protocol, lentivirus was added to the cells and stably transfected cell clones were selected using the limited dilution method. Finally, FSTL3 expression in cells was confirmed by Western blotting.

### RNA interference

The TfR1 siRNA sequence was 5’-CGGUGAUCAUAGUUGAUAA-3’ and 5’-CGUGCUACUUCCAGACUAA-3’, and the scramble control was 5’-UUCUCCGAACGUGUCACGUTT-3’. TfR1 siRNA (Gene Create, Wuhan, China) at a final concentration of 50 nM was transfected into cancer cells using Lipofectamine™ 2000 (Invitrogen Life Technologies, #11668030). Cells were collected for further studies 48 h after transfection.

### Generation of stable cell lines

CRISPR/Cas9-mediated targeting of the TfR1 gene was accomplished using single-guide RNAs (sgRNAs) delivered via the LentiCRISPR v2-Puro vector. The targeted sequence (Tsingke Biotechnology, Beijing, China) was 5’- CTACTTGGGCTATTGTAAAG-3’ for sgTfR1, and the scramble control was 5’-GGTTCTCCGAACGTGTCACGT-3’. TfR1 sgRNA was transfected into cancer cells using Lipofectamine™ 2000. To select stable transfected cell lines, 2 µg/mL puromycin (Solarbio, Beijing, China) was added for a duration of two weeks.

### Wound-healing and invasion assays

For the wound-healing assay, cells (70–80% confluent) were scraped smoothly to generate a gap and were exposed with or without recombinant human FSTL3 (rhFSTL3, 300 ng/mL, MedChem Express, Monmouth Junction, USA) and treated with or without an AKT inhibitor (MK-2206 dihydrochloride, 10μmol/L, MedChem Express) for 24 h. Images were captured at 0 and 24 h. For the invasion assay, 1 × 10^5^ cells were seeded into Matrigel-coated transwell inserts (8-μm pore size) with different treatments in serum-free medium and medium with 20% FBS in the lower chamber acting as a chemoattractant. After 48 h, cells that did not invade were wiped out with cotton swabs, and those that invaded into the underside of membranes were fixed in methanol, stained with 0.5% crystal violet, and then scored under a microscope. Three fields of each sample were captured to evaluate the average number of invaded cells.

### VM detection in tumor tissue specimens

Periodic acid–Schiff (PAS) staining was utilized to detect VM in tumor tissues [13, 22, 70]. The CD31 antibody (Abcam, #ab182981, 1:100, RRID: AB_2920881) and PAS staining are the current morphological foundation for VM development in malignant tumors. VM structure is determined by (1) the absence of vascular ECs on blood vessel walls, (2) the presence of tumor cells around vascular-like channels, (3) PAS^+^/CD31^−^ staining, and (4) erythrocytes in VM channels [7]. VM may be directly measured in tumors using these criteria.

### Vascular-like structure formation

The VM assay was performed using Matrigel (Corning, NY, USA), which was thawed at 4 °C overnight. And 50 μL of de-thawed Matrigel was used to coat each well in a 96-well plate and was allowed to polymerize for 1 h at 37 °C. After equilibrating the gel with complete growth medium, 1 × 10^5^ colon cancer cells (HCT116 and HT-29) and 5 × 10^4^ HUVEC were seeded in each well and incubated for approximately 12 and 4 h, respectively. The VM assay was performed with or without rhFSTL3 (300 ng/mL) and treated with or without a MK-2206 dihydrochloride (10μmol/L) for 24 h. Tube formation was observed under a phase-contrast microscope; three fields of each sample were captured to evaluate the average number of VM formation.

### Immunohistochemistry (IHC) staining

Anti-FSTL3 antibody (Abcepta, #AP12300b, 1:100, RRID: AB_10821490), anti-pAKT (Ser473) (Cell signaling technology, #4060 T, 1:200, RRID: AB_2315049), anti-VE-Cadherin (Wanleibio, #WL02033, 1:100, RRID: AB_3076321), and Ki67 (Abcam, #ab15580, 1:200, RRID: AB_443209) were used for IHC with a 2-step protocol [71]. The IHC score was assessed using a semiquantitative method based on the grade categories for the number of stained positive cells and the staining intensity, as previously reported [72]. The quantification process involved assessing the number of positively stained cells out of 100 cells in each of five selected high-power fields (×400 magnification) to determine the percentage of positively stained cells per section. The percentages were categorized as 0% (grade 0), 10–25% (grade 1), 26–50% (grade 2), 51–75% (grade 3), and 76–100% (grade 4). Staining intensity was further classified into grades 0, 1, 2, and 3, corresponding to no stain, light brown, brown, and dark brown, respectively. The IHC score was blindly reviewed by two pathology experts independently. High expression of FSTL3 was defined as an IHC score ≥ 1. The internal organs (heart, liver, spleen, lungs, and kidneys) were collected for pathologic staining.

### Masson’s staining

The procedure involved deparaffinization and rehydration of paraffin-embedded sections, followed by sequential staining with the provided solutions. After staining, the sections were dehydrated, cleared, and mounted with neutral gum. Under the microscope, collagen fibers appeared blue, while muscle fibers, fibrin, and red blood cells were stained red.

### Immunofluorescence (IF) staining

Cell slides or tissue sections (after tissue dewaxing and antigen repair) were prepared. Following the manufacturer’s instructions, IF staining was performed using the IHC kit and TUNEL detection kits (Beyotime) with the primary antibody incubating at 4 °C overnight, including anti-FSTL3 (Abcepta, #AP12300b, 1:100, RRID: AB_10821490), anti-FAP (Cell Signaling Technology, #66562, 1:1000, RRID: AB_2904193), anti-α-SMA (Santa Cruz, #sc-32251, 1:100, CA, USA, RRID:AB_262054), and anti-TfR1 (Proteintech, #10084-2-AP, 1:400, RRID: AB_2240403). The second antibody was incubated, and the slices were sealed using a DAPI (Servicebio, Wuhan, China) sealing agent. The reconstruction of the section images was based on the Virtual Microscope Software Slide Viewer 2.5 3D Histech®, and the fluorescence intensity was analyzed using ImageJ software (RRID: SCR_003070).

### In vivo anti-tumor efficacy in subcutaneous and in situ tumors

5–6-week-old male BALB/c nude mice were purchased from the Animal Experiment Center of Southeast University. Animals were assigned to experimental groups using simple randomization. For tumor xenografts, the mixture of HCT116 cells (1 × 10^6^) and shFSTL3 PCAF cells (3 × 10^6^) was mixed with Matrigel up to 100 µL and was subcutaneously injected into the right flank of an experimental group of each nude mouse, while the controlled group consists of a mixture of HCT116 cells (1 × 10^6^) and shControl PCAF cells (3 × 10^6^). Tumor diameters were measured using a caliper every two days, and the tumor volume was calculated using the formula: *V* (mm^3^) = (width)^2^ × length/2. Orthotopic models were constructed by injecting the same number of mixed cells into the cecum per mouse as above. The above experimental findings were indirectly verified by the results of an in vivo imaging system (IVIS, Tanon ABL-X5, Shanghai, China) assay. All experimental procedures were approved by the Institutional Animal Care and Use Committee of Southeast University.

### The highest single agent (HAS) synergistic scores

Synergistic scores of drug combinations were analyzed using SynergyFinder 3.0 (RRID: SCR_019318) [73]. SynergyFinder (https://synergyfinder.fimm.fi) was used to analyze and visualize FSTL3-targeting antibodies (aFSTL3) and bevacizumab combination responses relating to vascular-like structure formation. HAS synergistic scores between −10 and 10 were considered additive effects, and scores above 10 were considered synergistic effects.

### aFSTL3 synthesis and purification

The aFSTL3, FSTL3-FC (biotin), and Activin A-mFC proteins were synthesized and purified by SGE BIOTECH (Suzhou, China). The specific sequences were listed in Table S6. Initially, the heavy and light chains of the aFSTL3 were cloned into the PTT5-human kappa CL and PTT5-human IgG1 CH vectors, while the Activin A and FSTL3 genes were fused with mouse and human Fc fragments and cloned into the pCDNA3.4 vector. Following plasmid extraction, we transfected 293F cells (RRID: CVCL_6642) with the plasmid DNA using TA-293 transfection reagent. After 24 h, a protein expression enhancer was added, and the cells were harvested six days post-transfection. The supernatant was then centrifuged and filtered to eliminate debris. For purification, Protein A affinity chromatography was employed to isolate Fc-tagged proteins. Subsequently, FSTL3-FC was biotinylated and further purified through ultrafiltration. The final step involved a sandwich ELISA to confirm the presence of biotinylated FSTL3-FC, demonstrating effective recognition by the aFSTL3 and Activin A-mFC. This comprehensive approach successfully generated aFSTL3 that effectively target FSTL3 for further analysis.

### Statistical analysis

All statistics are expressed as mean ± standard deviation (SD). Multiple-group comparisons were performed by two-sided *t* test or one-way ANOVA followed by a Tukey correction to compare each group. The Chi-Squared test was applied for categorical data. The Kaplan-Meier method with the Log-rank test or Gehan-Breslow-Wilcoxon test was used to compare survival time between different groups. The hazard ratios (HR) and 95% confidence intervals (CI) for prognostic factors were calculated using a Cox regression model. Pearson correlation analysis was applied for the relationship between two indicators. The GraphPad Prism 9 software (GraphPad Software Inc., USA, RRID: SCR_002798) were used for statistical analysis. And the statistical significance was set to *, *p* < 0.05; **, *p* < 0.01; and ***, *p* < 0.001. All in vitro experiments were performed with at least three biological replicates.

## Results

### FSTL3 as a prognostic factor in CCAFs links to VM formation in colon cancer

To investigate the link between CCAFs and VM formation in the TME of colon cancer, we conducted WGCNA [62]. Using optimal β values of 3 for the TCGA-COAD cohort and 4 for the GSE39582 cohort, we identified 15 and 17 significant co-expression gene modules with TOMplot, respectively (Fig. S1A, B). The significance of the epigengene between the turquoise module genes and the CCAFs infiltration enrichment score was higher than that of other module genes in both TCGA_COAD and GSE39582 cohorts (Fig. S1C, D). This suggests that genes in the turquoise module are strongly linked to CCAF presence in the TME of colon cancer.

**Fig. 1:**
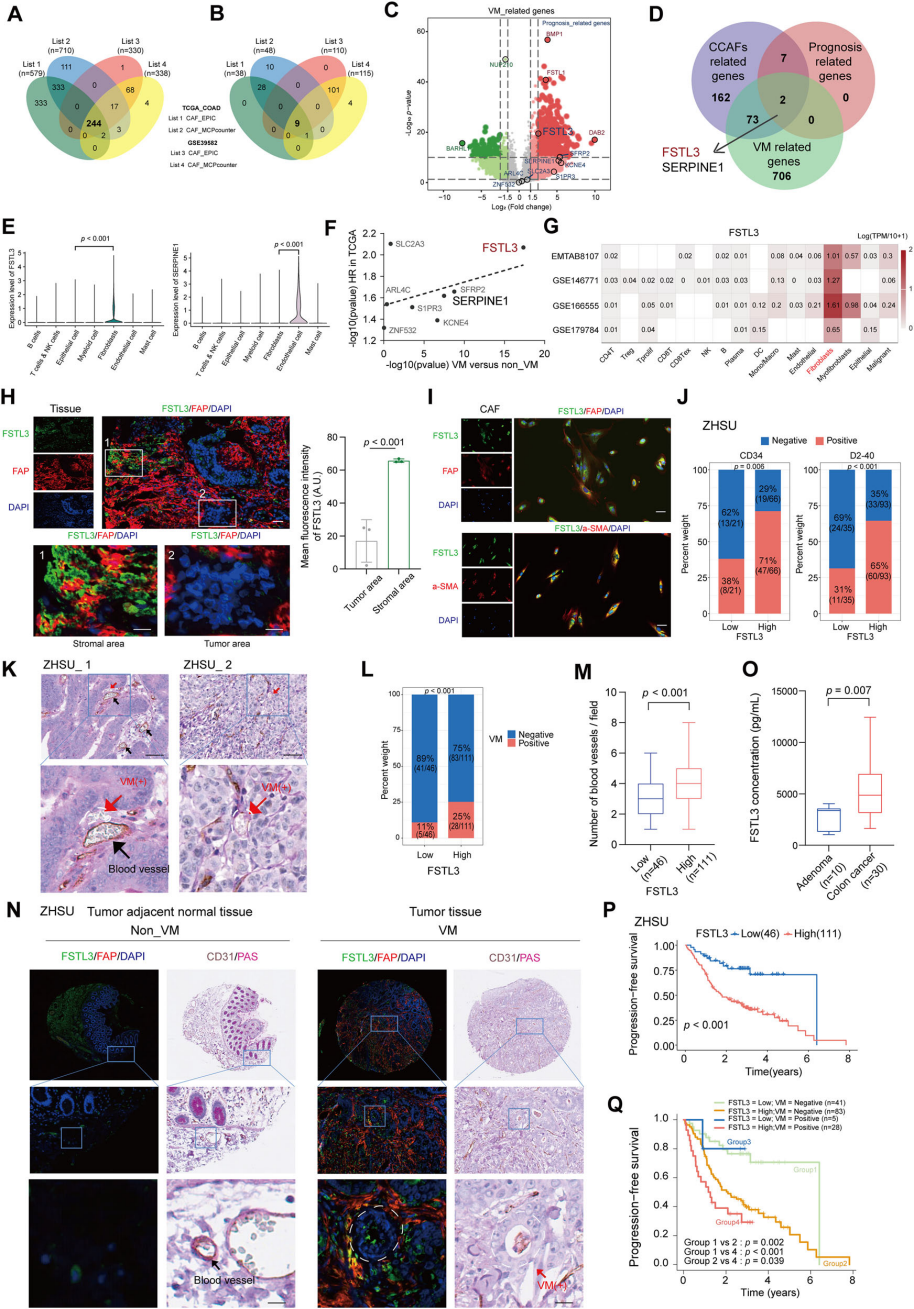
FSTL3 as a prognostic factor predominantly expressed in CCAFs and associated with VM formation in colon cancer. **A**, **B** Venn diagram showed the intersection of fibroblast-related genes (**A**) and prognosis-related genes (**B**) by CAF_EPIC and CAF_MCPcount methods in TCGA and GSE39582 cohorts. **C** Volcano plot showed genes significantly upregulated (Red labeled) and downregulated (Green labeled) in VM group using data from GSE240789 cohort. **D** Venn diagram showed the intersection of fibroblast-related genes, prognosis related genes and VM-related genes. **E** Violin plot showed the expression levels of FSTL3 and SERPINE1 different cell subtypes in GSE166555 cohort. **F** The significance distribution of gene for the difference between non_VM and VM tissues, as well as for the prognostic analysis. **G** The heatmap displayed the expression of FSTL3 among different cell types in four different scRNA-seq cohorts on the TISCH online database. **H** IF staining of FAP and FSTL3 in colon cancer tissues and the quantification of FSTL3 intensity in stroma area and tumor area (Scale bar =100 μm, n = 3, biologically independent samples). **I** IF staining of FAP, a-SMA, and FSTL3 in primary CCAFs (Scale bar =100 μm). **J** Histogram comparing the proportion of CD34 (*n* = 87), D2-40 (*n* = 128) positivity between the high- and low- expression groups of FSTL3. **K** CD31/PAS IHC staining for the tissue from ZHSU. The black arrow represents VM (−) which is CD31^+^PAS^-^, and the red arrow represents VM (+) which is CD31^-^PAS^+^. Both tubes contain red blood cells (Scale bar =100 μm, *n* = 157). **L**, **M** Histogram comparing the proportion of VM positivity (**L**) and the blood vessel intensity (**M**) between the high- and low-expression groups of FSTL3. **N** IF staining of FAP and FSTL3, along with CD31/PAS IHC staining, was performed on colon cancer tissue and its adjacent tumor tissue, two adjacent slices from a continuous section were used for the IF and IHC staining to ensure the results correspond to the same area, the black arrow indicates CD31^+^ blood vessels, while the red arrow and white dashed line represent VM; Scale bar =10 μm. **O** Bar graph comparing plasma FSTL3 protein levels in patients with colon cancer (*n* = 30) and colon adenomas (*n* = 10). **P** Kaplan–Meier survival curves for PFS in ZHSU cohort stratified by FSTL3 expression levels; **Q** Kaplan–Meier survival curves on PFS of the ZHSU cohort stratified by FSTL3 expression and VM status. All statistics are expressed as mean ± SD. Statistical significance was calculated by two-tailed *t* test in (**E**), (**H**), (**M**), and (**O**). The Chi-Squared test was applied for categorial data in (**J**) and (**L**). Statistical analysis of survival time was performed using log-rank tests in (P) and Gehan–Breslow–Wilcoxon test in (**Q**).

Univariate Cox regression analysis for OS and progression-free survival (PFS) indicated that the CAF_EPIC and CAF_MCPcount enrichment methods were top prognostic risk factors in both cohorts (Fig. S1E–G). Hub genes from the turquoise module, identified using the criteria |GS | > 0.5 and |MM| > 0.6 (Fig. S1H, I and Table S7**)**, yielded 244 common hub genes (Fig. 1A). Further univariate Cox regression analysis revealed nine prognosis-related genes (HR > 1, *p* < 0.05) from these 244 hub genes (Fig. 1B and Table S8). To identify VM-related genes, we conducted DGE analysis between non-VM and VM samples using data from the GSE240789 cohort [63], identifying 781 upregulated genes associated with VM formation (Fig. 1C and Table S9). The intersection of the 244 hub genes, the nine prognosis-related genes, and the VM-related gene highlighted FSTL3 and SERPINE1 as key CCAF-related genes involved in VM formation. (Fig. 1D).

FSTL3 and SERPINE1 were further examined for their expression across different cell types using the published colon cancer scRNA-seq cohort (GSE166555). FSTL3 was predominantly expressed in fibroblasts, whereas SERPINE1 showed higher expression in endothelial cells (Fig. 1E and Fig. S2A–D**)**. Moreover, FSTL3 levels were significantly elevated in tumor-associated fibroblasts compared to their normal counterparts (Fig. S2E). Among the examined genes, FSTL3 showed the strongest correlation with both poor prognosis and VM-related differential expression (Fig. 1F), suggesting its potential as a marker for VM and a contributor to poor prognosis in colon cancer.

Previous studies have primarily focused on tumor-expressed FSTL3 and its autonomous or non-autonomous functions [40, 43, 74–76]. To further validate FSTL3’s expression pattern, we examined several published scRNA-seq databases. Consistent with GSE166555, FSTL3 was predominantly expressed in fibroblast cells of colon cancer (Fig. 1G and Fig. S2F). Analysis of the CCLE database confirmed that FSTL3 expression was significantly higher in CCAFs compared to colorectal cancer cells (Fig. S2G). Moreover, RNA-seq data from TCGA_COAD and GSE39582 cohorts revealed that FSTL3 expression strongly correlated with fibroblast markers (FAP, α-SMA) (Fig. S2H) and showed a modest inverse correlation with epithelial markers (CEA, CK20, EpCAM) (Fig. S2I), reinforcing the fibroblast-specific expression of FSTL3.

To further validate these findings, we collected tumor and adjacent normal tissue samples from 157 colon cancer patients at ZHSU cohort, and performed IF staining for FAP and FSTL3. Consistently, we observed that FSTL3 was highly expressed, especially in the stromal areas of tumors, and co-localized with FAP staining (Fig. 1H). Further, FSTL3 also co-localized with FAP and α-SMA in primary CCAFs derived from colon cancer (Fig. 1I).

CCAFs exhibit substantial heterogeneity [31, 52], and it was important to determine which subpopulations express FSTL3. We examined the GSE166555 cohort, where CAFs were divided into seven distinct subpopulations. FSTL3 was most highly expressed in the “Vascular CAFs” and “Tumor-like CAFs” subpopulations, which are known to play key roles in angiogenesis and tumorigenesis [52] (Fig. S2J–L).

To explore the role of FSTL3 in colon cancer progression, we performed IHC on colon cancer tissues from ZHSU. Our results revealed that patients with high FSTL3 expression are more likely to be CD34 and D2-40 positive, indicating lymphatic and blood vascular invasion [74, 75], respectively (Fig. 1J). This supports the idea that FSTL3 is positively correlated with lymphatic invasion, metastasis, and poor prognosis, as previously reported [43, 44]. Additionally, co-staining of PAS and CD31 revealed that VM formation was 2.3 times more prevalent in tumors with high FSTL3 expression (Fig. 1K, L). Similarly, the CD31^+^ blood vessel intensity was higher in the FSTL3-positive group than in the FSTL3-negative group (Fig. 1M). Moreover, tumor tissues exhibited significantly higher levels of both FAP and FSTL3 staining and VM formation compared to adjacent normal tissues. Interestingly, FSTL3^+^ CCAFs were found to co-localize with VM regions, suggesting that FSTL3^+^ CCAFs may play a critical role in promoting cancer cell transformation and VM formation (Fig. 1N). Additionally, plasma FSTL3 levels were significantly higher in colon cancer patients compared to those with adenoma (Fig. 1O), further suggesting its potential as a diagnostic marker for colon cancer.

Furthermore, survival analysis revealed that patients with high FSTL3 expression had significantly worse OS and PFS compared to those with low expression in the TCGA-COAD, GSE39582, and ZHSU cohorts (Fig. 1P and Fig. S3A, C, D). Notably, in the ZHSU cohort, patients were stratified into four groups based on FSTL3 expression and VM status. The results indicated that patients with high FSTL3 and VM-positive status had the worst prognosis, further suggesting that FSTL3, combined with VM status, could serve as a powerful prognostic marker in colon cancer (Fig. 1Q and Fig. S3B).

### CCAFs promote VM formation and metastasis via upregulating FSTL3

**Fig. 2:**
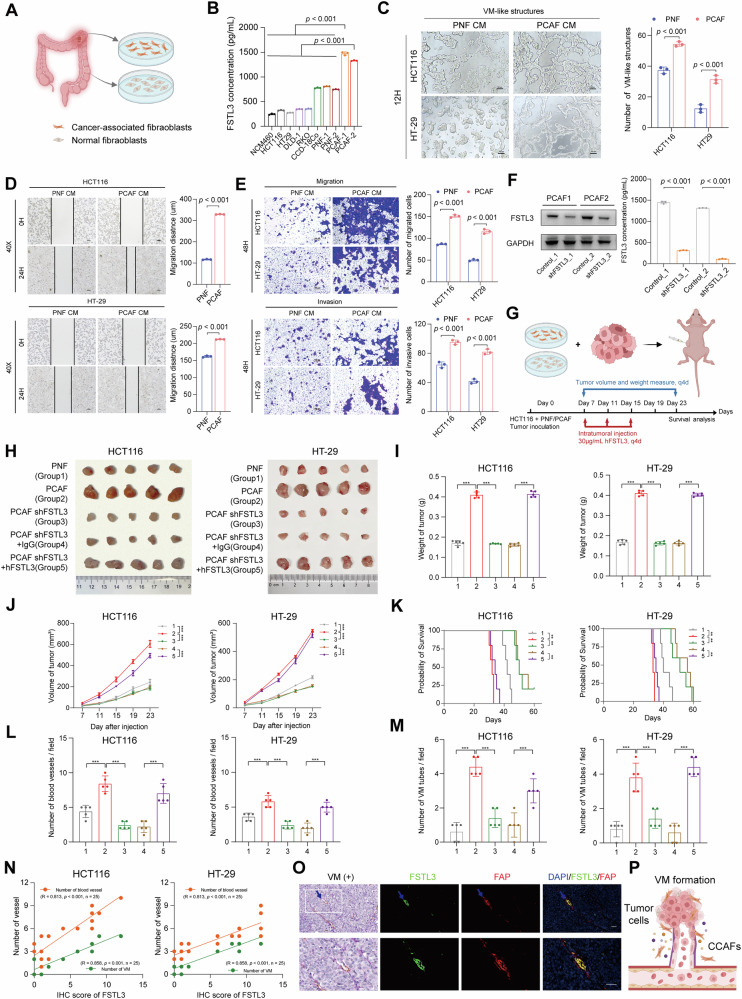
CCAFs significantly promote VM formation and malignancy of colon cancer via upregulating FSTL3. **A** Schematic illustration of the establishment of PNF and PCAF primary cell line. **B** FSTL3 levels in the supernatant of different cell line measured by ELISA. **C** Tube-like structure formation of HCT116 and HT-29 cells treated with PNF or PCAF conditioned medium for 12 h and its quantification, Scale bar =100 μm, *n* = 3. **D** Scratch assay to test migration ability of HCT116 and HT29 treated with conditioned medium of PNF or PCAF, Scale bar =100 μm, *n* = 3. **E** Transwell assay to test the migration and invasion ability of HCT116 and HT29 cell treated with conditioned medium of PNF or PCAF, Scale bar =100 μm, *n* = 3. **F** Western blot and ELISA confirming the knocking down of FSTL3 in PCAF cells through shRNA. **G** Schematic illustration of the establishment of the in vivo tumor model to confirm the function of FSTL3 in CCAF. **H** Images of HCT116 and HT-29 xenograft tumors at study end, *n* = 5/group. **I** Tumor weight of HCT116 and HT29 at study end *n* = 5/group. **J** Tumor growth curve of HCT116 and HT-29 in each group. **K** Mice survival curve of different groups, *n* = 5/group. **L**, **M** Quantification of blood vessels (**L**) and VM numbers (**M**) in three random fields for each sample in different groups, *n* = 5 samples/group. **N** The correlation between the FSLT3 level based on IHC staining and the number of blood vessels and VM tubes in HCT116 and HT-29 model. **O** CD31/PAS IHC staining and IF staining for FAP and FSTL3, two adjacent slices from a continuous section were used for the IF and IHC staining to ensure the results correspond to the same area. The blue arrow represented VM (+), tubes contain red blood cells. Scale bar =100 μm. **P** Schematic illustration of the VM; BioRender.com was used to create (**A** and **P**). All statistics are expressed as mean ± SD. *p < 0.05, **p < 0.01, and ***p < 0.001. Statistical significance was calculated by two-tailed *t* test in (**B**–**F**), and one-way ANOVA with the Tukey post hoc test in (**I**), (**J**), (**L**) and (**M**). Statistical analysis of survival time was performed using log-rank tests in (**K**). Pearson correlation analysis was applied for the relationship between two indicators in (**N**).

To further explore the role of CCAF-derived FSTL3 in VM formation and colon cancer malignancy, we generated primary fibroblast cell lines from colon cancer (PCAF) and adjacent normal tissue (PNF) (Fig. 2A). ELISA analysis showed that FSTL3 levels were significantly higher in the conditioned medium (CM) from PCAF compared to normal colon epithelial cells, colon cancer cell lines, and PNF (Fig. 2B). Culturing colon cancer cells with CM from PCAF significantly enhanced tube-like structure formation in both HCT116 and HT-29 cells (Fig. 2C) and promoted migration and invasion (Fig. 2D, E). Given the positive correlation between FSTL3 and blood vessel formation in colon cancer patients, we also examined the effect of PCAF-derived CM on HUVECs. The results revealed that PCAF CM significantly enhanced the ability of HUVECs to form vessel-like structures (Fig. S4A).

Mass spectrometric analysis of CCAF secretomes from other report revealed that FSTL3 was abundant and may promote lymph-angiogenesis and other malignant behaviors in tumors [77, 78]. To confirm that the promotion of VM and blood vessel formation by CCAFs is mediated by FSTL3, we firstly exogenously added rhFSTL3. This treatment significantly enhanced tube-like structure formation and increased migration and invasion in both HCT116 and HT-29 cells (Fig. S4B–F). Similarly, rhFSTL3 also promoted tube-like structure formation in HUVECs (Fig. S4G).

Next, to investigate the role of FSTL3 secreted by PCAF in tumor progression, we knocked down FSTL3 in PCAF, which resulted in a substantial decrease in FSTL3 secretion (Fig. 2F and Fig. S5A). When HCT116 cells were co-injected with PCAF or FSTL3-knockdown PCAF into nude mice, tumor growth was significantly reduced in the FSTL3-knockdown group, and mouse survival was prolonged (Fig. S5B–D). Both VM formation and blood vessel density in tumors were also reduced (Fig. S5E, F). In comparison to PNF, PCAF exhibited significantly more advanced pro-tumor growth in both HT-29 and HCT116 models in vivo. However, this pro-tumor growth effect was markedly diminished when FSTL3 was knocked down in PCAF. Furthermore, exogenous addition of rhFSTL3 to the FSTL3-knockdown PCAF group significantly reversed the effects, promoting colon tumor growth and decreasing mouse survival (Fig. 2G–K). IF staining and IHC-PAS co-staining further revealed that FSTL3 is critical for PCAF-induced blood vessel and VM structure formation in tumors (Fig. 2L, M and Fig. S5G). Notably, the protein expression level of FSTL3 was significantly positively correlated with the number of blood vessels per unit field of view and the number of VM formations (Fig. 2N). Similar to what we observed in human patients, a cluster of FSTL3^+^ CAF cells were observed surrounding the VM in the tumor (Fig. 2O, P). This suggests that FSTL3 secreted by CAFs plays a crucial role in promoting colon cancer cell transformation and VM formation likely through direct interactions with cancer cells.

### Hypoxia-induced FSTL3 expression and ECM remodeling promote EET transformation in colon cancer cells

**Fig. 3:**
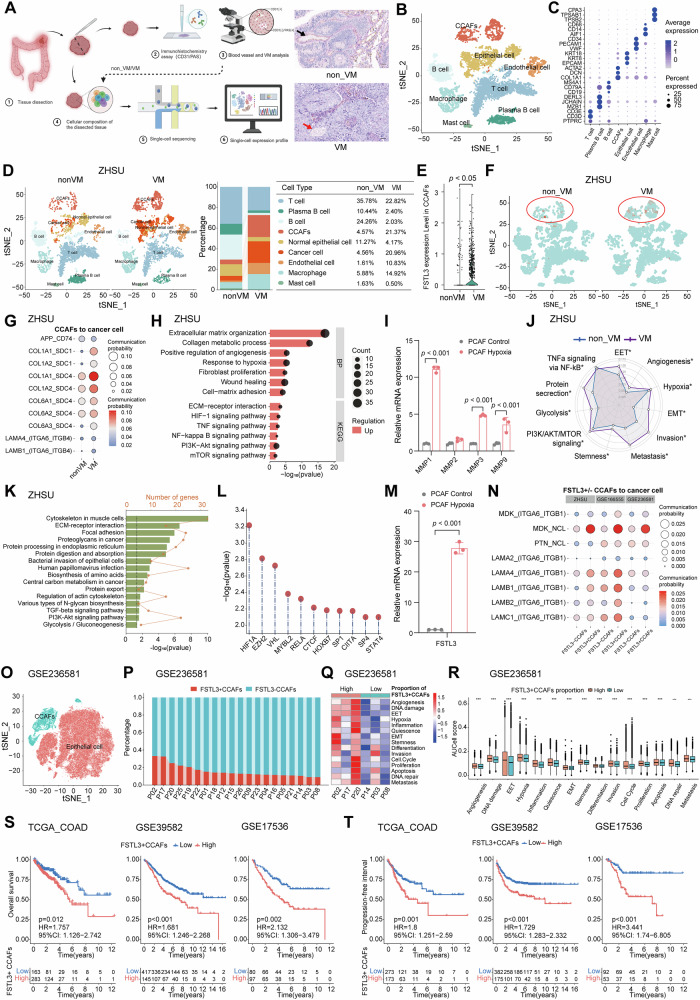
scRNA-seq highlights the role of hypoxia in driving FSTL3 expression and enhancing ECM remodeling in VM_CCAFs to promote colon cancer cell EET transformation. **A** The study design for scRNA-seq and CD31/PAS IHC staining for non-VM and VM samples, scRNA-seq was conducted for the samples confirmed to be VM positive or negative; CD31 and PAS IHC staining for the tissue. The black arrow represents VM (−) which is CD31^+^PAS^-^, and the red arrow represents VM (+) which is CD31^-^PAS^+^. Both tubes contain red blood cells (Scale bar =100 μm). **B** tSNE plot of cells from non_VM and VM patients. **C** Bubble plot showed the differential marker genes in eight subtypes. **D** The tSNE plots and histograms showing cell clusters and their ratios. **E** Violin plot showing FSTL3 expression across different cell types in non-VM and VM sample. **F** tSNE plot to show FSTL3 level in non-VM and VM sample. **G** Ligand–receptor interaction pattern analysis between CCAF and cancer cells in nonVM and VM samples. **H** GO and KEGG pathway enrichment analysis using gene upregulated in VM. **I** qPCR analysis for MMP1, MMP2, MMP3, MMP9 gene expression under control or hypoxia conditions. **J** The AUCell score of ten-hallmark related pathways using all the cells in the non_VM and VM samples. **K** KEGG pathway enrichment analysis for the gene upregulated in FSTL3^+^ CCAF compared to FSTL3^-^ CCAFs. **L** TRRUST Transcription Factor Enrichment analysis using the top 250 genes upregulated in FSTL3 + CCAF, ranked by P-value, with a log2 fold change greater than 0.25. **M** qPCR analysis of FSTL3 transcription in PCAF under control or hypoxia conditions. **N** Analysis of ligand-receptor interaction patterns between cancer cells and FSTL3^+^ CCAFs or FSTL3^-^ CCAFs in ZHSU, GSE166555 and GSE236581 cohorts. **O** tSNE map of epithelial cells and fibroblasts in the GSE236581 cohort, *n* = 20. **P** The proportion of FSTL3^+^ CCAFs among each patient. **Q** Heatmap of AUCell scores of 15 gene sets for the epithelial cell from top three proportion FSTL3^+^ CCAFs patients and bottom three FSTL3^+^ CCAFs patients. **R** The bar plot showed the AUCell score of signaling pathway in FSTL3^+^ CCAFs high proportion and low proportion groups. **S**, **T** Kaplan–Meier survival curves of OS and PFS in TCGA_COAD, GSE39582 and GSE17536 cohorts stratified by low and high FSTL3^+^ CCAFs signature. All statistics are expressed as mean ± SD. *p < 0.05, **p < 0.01, and ***p < 0.001. Statistical significance was calculated by two-tailed *t* test. The Kaplan–Meier method with the Log-rank test was used to compare survival time between two groups.

To investigate how FSTL3 affects CCAFs’ function and to explore the differences between CCAFs in colon tumors from verified VM and non-VM patients, we collected tumor tissue from one verified VM and one verified non-VM patient for scRNA-seq analysis. To achieve this, the tumor tissue from each patient was first processed for CD31/PAS IHC co-staining, scRNA-seq was only processed when the VM status was confirmed (Fig. 3A). Following quality control, we obtained 9,901 high-quality cells, which were clustered and identified as T cells (29.7%), Plasma B cells (6.7%), B cells (13.9%), CCAFs (12.4%), epithelial cells (20.2%), ECs (5.9%), macrophages (10.1%), and mast cells (1.1%) using Single R as previous reported [50] (Fig. 3B, C and Fig. S6A–C). The “copyKAT” tool was then used to objectively distinguish epithelial cells as normal or cancer cells. Notably, VM tumors exhibited a higher proportion of cancer cells, CCAFs, and macrophages, while populations of normal epithelial cells, as well as T and B cells, were reduced (Fig. 3D and Table S10). Consistent with the findings above, FSTL3 expression was markedly elevated in CCAFs, particularly in VM patients (Fig. 3E, F and Fig. S6D).

CellChat analysis revealed that CCAFs from VM patients exhibited significantly increased interactions with other cell types, including cancer cells, compared to non-VM CCAFs (Fig. S6E). Specifically, the ligand-receptor interaction patterns of COL6A1/2/3 and COL1A1/2 on CCAFs with SDC4 and SDC1 on cancer cells were markedly enhanced in VM patients (Fig. 3G). These collagen interactions are key drivers of ECM remodeling in colon cancer, as expressed by CCAFs [31]. Additionally, the interactions between LAMA4 and LAMB1 expressed on CCAFs with ITGA6_ITGB4 expressed on cancer cells were enhanced in VM patients, indicating stronger physical interactions between CCAFs and cancer cells (Fig. 3G). On the other hand, APP-CD74 interactions were decreased in VM patients (Fig. 3G). Furthermore, GO and KEGG analysis of genes upregulated in VM CCAFs revealed enrichment in matrix remodeling and vasculature formation pathways, such as extracellular matrix organization, collagen metabolic processes, and cell-matrix adhesion (GO analysis), as well as ECM-receptor interaction (KEGG) (Fig. 3H). Specifically, ECM-related genes, such as MMPs, collagens, and cytokines CXCL1-3, as well as the inflammatory gene IL1B, were significantly upregulated in VM CCAFs (Fig. S6F). Collectively, these findings emphasized the significant enhancement of ECM remodeling in VM CCAFs.

Further investigation revealed that hypoxia and HIF-1 signaling pathways were enriched in VM CCAFs (Fig. 3H). Previous studies have shown that hypoxia plays a crucial role in determining CAF differentiation and function [31, 79, 80]. To assess whether hypoxia induces ECM remodeling-related gene expression in CCAFs, we treated PCAF under hypoxic conditions and observed significant upregulation of MMP1/3/9 by qRT-PCR (Fig. 3I). Furthermore, AUCell scores for gene sets associated with EET and hallmark-related pathways identified in the literature [54, 55] revealed significant upregulation of pathways related to EET, angiogenesis, hypoxia, EMT, invasion, metastasis, and stemness in VM patients (Fig. 3J and Table S11).

To investigate the role of FSTL3 in CCAFs, we compared FSTL3^+^ and FSTL3^-^ CCAFs. Notably, genes highly expressed in FSTL3^+^ CCAFs were significantly enriched in pathways related to ECM-receptor interaction and the PI3K-Akt signaling pathway (Fig. 3K). To identify potential drivers of this difference, TRRUST_Transcription_Factors Enrichment analysis was performed using genes upregulated in FSTL3^+^ CCAFs. HIF1A emerged as a top significant factor across multiple cohorts, including ZHSU, GSE166555, and GSE236581 (Fig. 3L and Fig. S6G, H). These findings suggest that hypoxia plays a pivotal role in driving FSTL3 expression in CCAFs. This was further confirmed by qRT-PCR analysis, which showed a significant increase in FSTL3 expression under hypoxic conditions (Fig. 3M).

Additionally, CellChat analysis revealed that FSTL3^+^ CCAFs exhibited a greater number and strength of interaction with other cell types, including cancer cells but not normal epithelial cells, compared to FSTL3^-^ CCAFs (Fig. S6I–K**)**. Specifically, the ligand-receptor pairs MDK-NCL, LAMA4 and LAMB1/2 with ITGA6_ITGB1 were significantly enhanced in FSTL3^+^ CCAFs compared to FSTL3^-^ CCAFs across all cohorts. MDK-NCL pairs between CAFs and cancer cells have been reported to drive immunosuppressive TME, promoting cancer progression [81, 82]. These enhanced interactions further revealed that FSTL3^+^ CCAFs have higher physical interaction probabilities with cancer cells (Fig. 3N).

To further explore how FSTL3^+^ CCAFs affect cancer cells, we analyzed data from 53 samples obtained from 20 patients with colon cancer, prior to immunotherapy, from the GSE236581 cohort (Fig. 3O). Samples were stratified based on the proportion of FSTL3^+^ CCAFs, resulting in high and low FSTL3^+^ CCAF proportion groups (Fig. 3P). AUCell scores for gene sets associated with angiogenesis, EET, EMT, and metastasis were significantly higher in the high-proportion group (Fig. 3Q, R). Furthermore, signaling pathways related to tumor progression were significantly enriched in patients with a higher proportion of FSTL3^+^ CCAFs. Next, we generated a FSTL3^+^ CCAF gene signature—which showed strong prognostic potential for OS and PFS in multiple cohorts, including TCGA_COAD, GSE39582, and GSE17536 (Fig. 3S, T).

### FSTL3 induces VM formation via activating AKT/mTOR/VE-Cadherin pathway

**Fig. 4:**
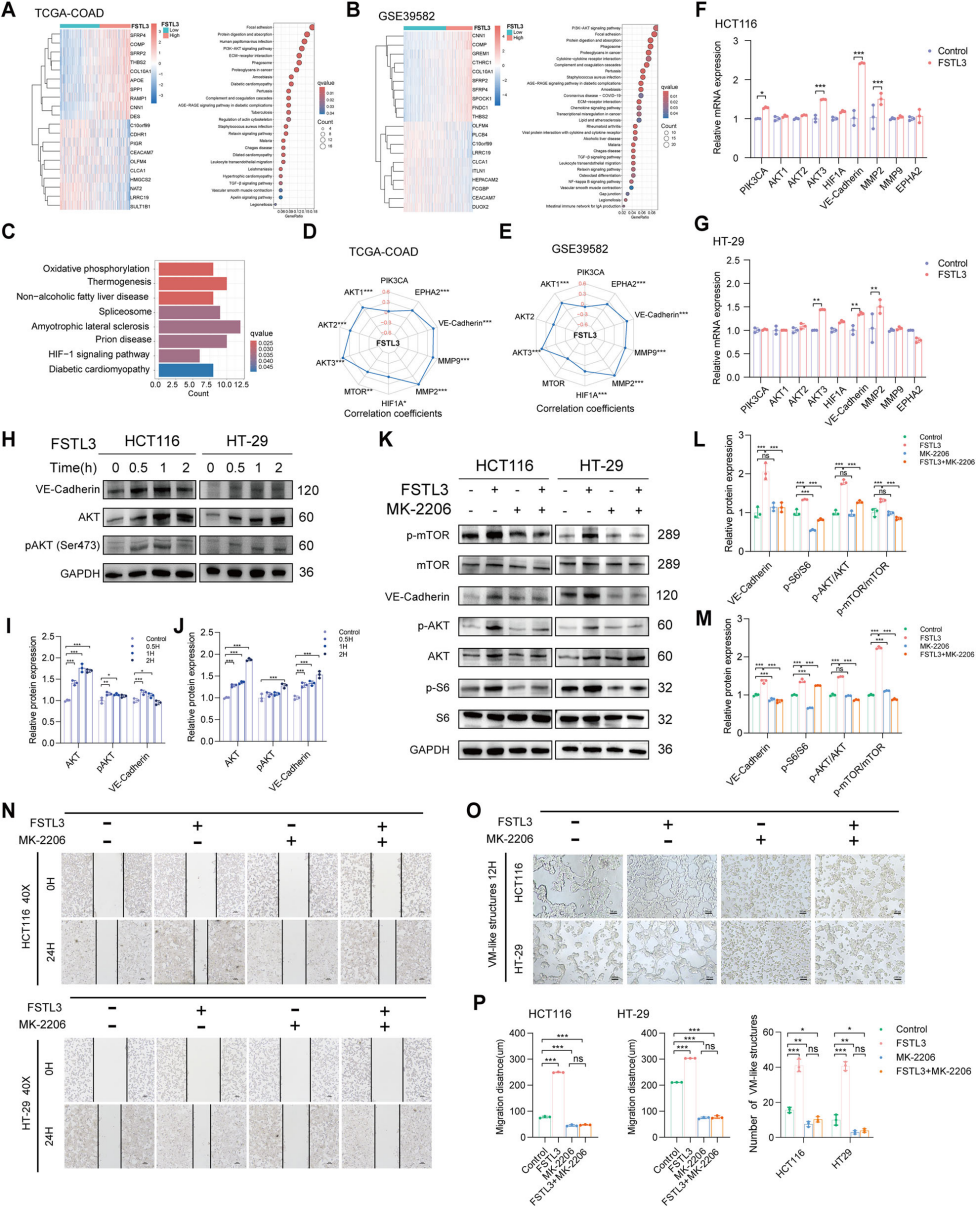
FSTL3 induces colon cancer cell transformation and VM formation via activating AKT/mTOR/VE-Cadherin cascade. **A**, **B** Heat map illustrating the top 10 differential genes in the transcriptome of FSTL3 high- and low-expression groups, along with KEGG enrichment analysis bubble charts of differentially expressed genes from the TCGA-COAD (**A**) and GSE39582 (**B**) cohorts; **C** KEGG enrichment analysis of differentially expressed genes in HCT116 cells with and without the addition of rhFSTL3. **D**, **E** Correlation analysis between FSTL3 and genes related to the angiogenesis pathway and EET. **F**, **G** qPCR analysis of genes in the PI3K/AKT signaling pathway and EET-related genes, *n* = 3. **H–J** Western blot analysis of indicated proteins (**H**) and their quantifications for HCT116 (**I**) and HT-29 (**J**) cells treated with 300 ng/mL rhFSTL3 at different time points. **K–M** Western blot analysis (**K**) of indicated proteins and their quantifications for HCT116 (**L**) and HT-29 (**M**) cells treated with or without rhFSTL3 and/or the AKT inhibitor MK-2206. **N** Representative images from a scratch assay demonstrating that MK-2206 effectively inhibited the enhanced migration of HCT116 and HT-29 cells induced by rhFSTL3, Scale bar =100 μm, *n* = 3. **O** Representative images of tube-like structure formation in HCT116 and HT-29 cells under different treatments, Scale bar =100 μm. **P** Quantification of migration ability and tube formation of HCT116 cells under different treatments, *n* = 3; All statistics are expressed as mean ± SD.*p < 0.05, **p < 0.01, ***p < 0.001, and ns, no significance. Pearson correlation analysis was applied for the relationship between two indicators in (**D**) and (E). Statistical significance was calculated by two-tailed *t* test in (**F**) and (**G**), and one-way ANOVA with the Tukey post hoc test in (**I**), (**J**), (**L**), (**M**) and (**P**).

To investigate the underlying mechanism of CCAFs-expressed FSTL3 in facilitating colon cancer progression, DGE analysis of the transcriptome was conducted on the FSTL3 high- and low-expression groups in TCGA-COAD and GSE39582, respectively (Fig. 4A, B). KEGG enrichment analysis revealed that differentially expressed genes were largely enriched in pathways associated with focal adhesion, protein digestion and absorption, and PI3K/AKT signaling (Fig. 4A, B). In addition, rhFSTL3 was exogenously added for the treatment of HCT116 cells, and RNA-seq was performed with the treated cells and the control cells. We found a significant differential expressed genes (*n* = 240) and an enrichment of HIF-1A signaling pathway (Fig. 4C). PI3K/AKT was reported to be essential for VM formation [7]. Consequently, we postulated that FSTL3 influences VM formation via the PI3K/AKT signaling pathway.

To confirm this, an initial step involved correlation analysis between the mRNA expression levels of FSTL3 and genes associated with the PI3K/AKT signaling pathway. FSTL3 was positively correlated with AKT in TCGA-COAD and GSE39582 cohorts (Fig. 4D, E). Furthermore, there was a consistent and substantial positive correlation between FSTL3 and marker genes associated with angiogenesis and EET, including HIF1A, MMP2, MMP9, VE-Cadherin, and EPHA2 (Fig. 4D, E). qRT-PCR confirmed that the treatment of rhFSTL3 on HCT116 and HT-29 cells led to a notable increase in the transcription of VE-Cadherin and MMP2 (Fig. 4F, G). The treatment of rhFSTL3 increased the protein levels of pAKT and VE-Cadherin in a time dependent manner (Fig. 4H–J), whereas the levels of MMP2 remained unchanged. To verify FSTL3 enhanced the protein expression levels of VE-Cadherin in colon cancer cells via activation of the AKT/mTOR signaling pathway, we treated the cancer cell with AKT inhibitor MK-2206. MK-2206 significantly blocked the activation of AKT/mTOR pathway and decreased the VE-Cadherin expression upregulated by rhFSTL3 (Fig. 4K–M). Furthermore, MK-2206 significantly attenuated FSTL3-induced cancer cell migration and vascular-like structure formation (Fig. 4N–P). Similarly, MK-2206 treatment also diminished the vascular-like structure formation of HUVEC cell induced by FSTL3 (Fig. S7A, B). Collectively, these data demonstrated that FSTL3 promotes VM development through activation of the AKT/mTOR/VE-Cadherin signaling pathway, thereby influencing the aggressive phenotypic characteristics of colon cancer.

### FSTL3 binds to TfR1 on colon cancer cells to promote VM and metastasis

**Fig. 5:**
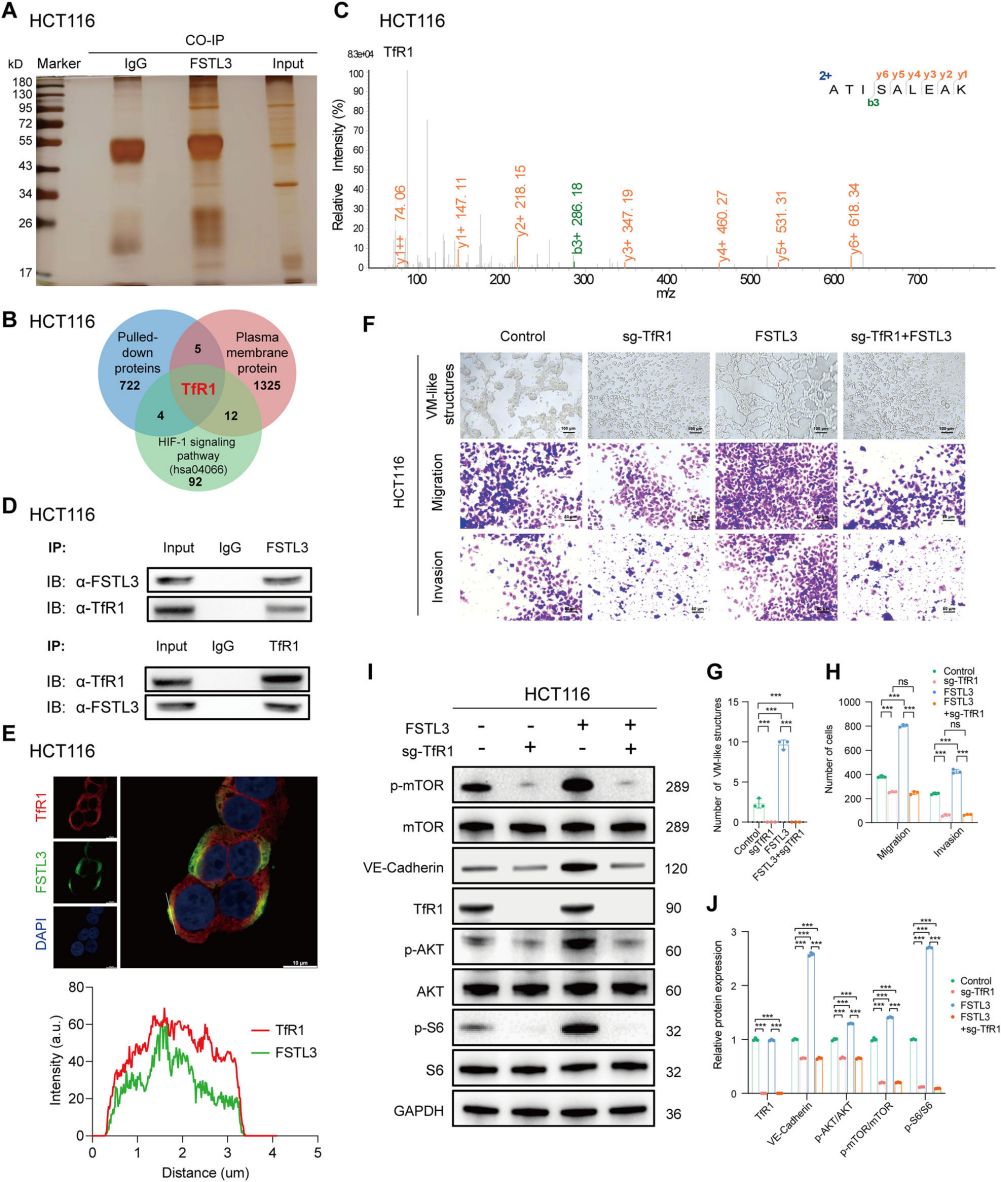
FSTL3 directly binds to TfR1 on colon cancer cells to promote VM and migration by inducing VE-Cadherin expression. **A** Silver staining of FSTL3-binding proteins following CO-IP. **B** Venn diagram showing the overlap between FSTL3-pulled down proteins, membrane proteins, and proteins associated with the HIF-1 signaling pathway. **C** The LC-MS/MS images of TfR1. **D** CO-IP followed by western blot analyses demonstrating the interaction between FSTL3 and TfR1. **E** Colocalization of FSTL3 and TfR1 by IF staining in HCT116 cells treated with 300 ng/mL exogenous rhFSTL3. The histogram below illustrated the change in fluorescent intensity along the line drawn in the merged images; Scale bar =10 μm. **F** Representative images of tube formation, cell migration, and cell invasion in vitro; Scale bar =100 μm, *n* = 3. **G**, **H** Quantification of tube formation (**G**) and migration or invasion ability of HCT116 cells (**H**), *n* = 3. **I, J** Western blot images (**I**) and quantification (**J**) illustrating protein expression levels in the TfR1/AKT/mTOR pathways following TfR1 knockout in HCT116 cells. All statistics are expressed as mean ± SD. *p < 0.05; **p < 0.01; ***p < 0.001; and ns, no significance. Statistical significance was calculated by one-way ANOVA with the Tukey post hoc test.

Next, to investigate how FSTL3 secreted by CCAFs activates the PI3K/Akt/VE-Cadherin pathway in cancer cells, we first performed an in vitro co-immunoprecipitation (Co-IP) experiment followed by mass spectrometry to identify membrane receptors that may interact with FSTL3 (Fig. 5A). Given that hypoxia plays a pivotal role in CCAF’s function and is essential for regulating FSTL3 expression, we performed a Venn analysis comparing pulled-down proteins with membrane proteins and those associated with the HIF-1 signaling pathway. This analysis identified the TfR1 as the only receptor with functional relevance to FSTL3 (Fig. 5B, C). TfR1, an iron-transferrin receptor encoded by the TFRC gene, mediates iron uptake and is overexpressed in rapidly dividing cells, such as cancer cells, due to their increased iron demand. Notably, TfR1 has been shown to be upregulated in various cancers [83, 84]. Co-IP experiments followed by Western blotting further confirmed the interaction between FSTL3 and TfR1 (Fig. 5D). IF staining also revealed that FSTL3 and TfR1 co-localized on the cell membrane of HCT116 cells (Fig. 5E). To evaluate the functional role of TfR1 in FSTL3-mediated effects, we knocked out or silenced TfR1 in HCT116 cells using CRISPR-Cas9 and siRNA approaches. In both TfR1 knockout and knockdown cells, FSTL3 stimulation failed to enhance VM-like structure formation or migration ability (Fig. 5F–H and Fig. S8A–C). Furthermore, upon TfR1 loss, FSTL3 stimulation did not lead to a significant upregulation of AKT/mTOR pathway components or VE-Cadherin expression (Fig. 5I, J and Fig. S8D, E). These findings suggest that TfR1 is a critical receptor for FSTL3 in regulating VM formation and metastasis in colon cancer.

Numerous studies have reported the autonomous role of FSTL3 in cancer cells, where it promotes cell proliferation and migration [43–45]. To investigate whether FSTL3 also exerts autonomous functions in CAFs, we silenced FSTL3 in CCAFs and concurrently treated them with aFSTL3 which synthesized as previously described [85]. Firstly, ELISA assays confirmed that aFSTL3 effectively binds to FSTL3 in vitro (Fig. S9). We then assessed the effects of this combined intervention on migration, invasion, and tube formation. Compared to the control group, aFSTL3 treatment alone did not affect these malignant phenotypes (Fig. S10A–C). However, FSTL3 knockdown in CCAFs significantly impaired migration and invasion, while having no effect on tube formation (Fig. S10A–C). Additionally, TfR1 expression in CCAFs was significantly lower than in colon cancer cells (Fig. S10D, E). Consistent with this, FSTL3 treatment failed to activate the AKT/mTOR/VE-cadherin signaling pathway in CCAFs (Fig. S10F).

### FSTL3 targeting combined with Bevacizumab improves prognosis

As a supplement to the angiogenesis pattern, VM formation contributes to tumor progression by improving blood supply and facilitating metastasis independently of conventional angiogenic mechanisms. Targeting VM-related molecules represents a promising strategy to enhance the efficacy of anti-angiogenic therapies. We performed a dose-dependent inhibition assay to assess tubular structure formation in HUVEC, HCT116, and HT29 cells, treated with varying combinations of aFSTL3 and Bevacizumab. The results revealed a strong synergistic effect in inhibiting HUVEC tube formation, with synergy scores (HAS) exceeding 10. A moderate synergistic effect was also observed in HCT116 and HT-29 cells, with HAS scores of 3.655 and 3.956, respectively (Fig. S11A–C).

**Fig. 6:**
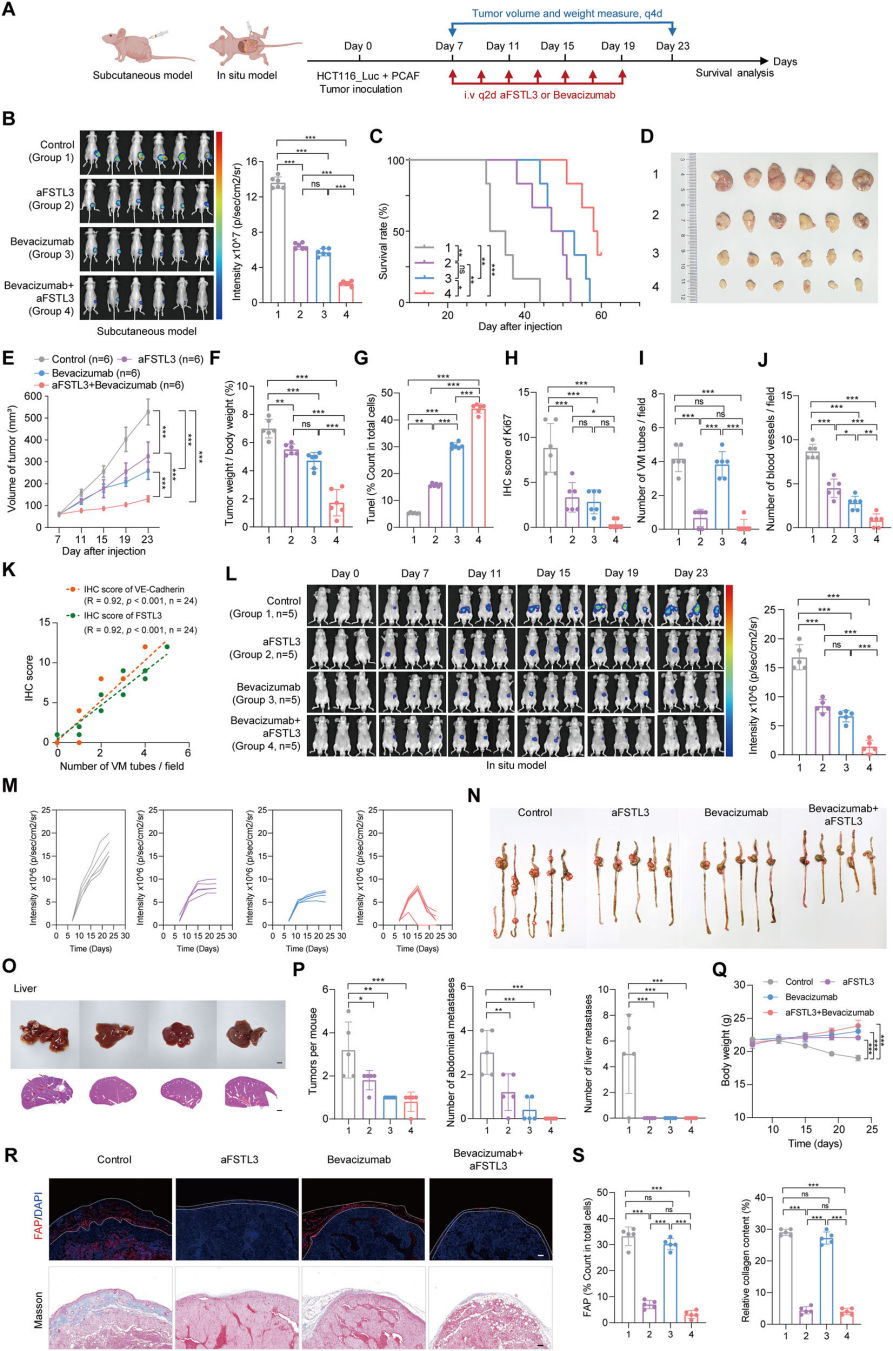
FSTL3 targeting in combination with Bevacizumab improves colon cancer prognosis. **A** Schematic illustration of the establishment of the in vivo tumor model, created by BioRender.com. **B** Bioluminescent IVIS in vivo imaging and quantitative results, *n* = 6. **C** Survival curve of mice with different treatment, *n* = 6. **D** Images of excised xenograft tumors at study endpoint, *n* = 6. **E** Tumor growth curve of different groups, *n* = 6. **F** The ratio of tumor weight to mice weight, *n* = 6. **G** Percentage of apoptotic cells detected by TUNEL assay, *n* = 6. **H** IHC intensity of Ki67, *n* = 6. **I**, **J** Quantification of VM numbers (**I**) blood vessels **(J**) in three random fields for each sample in different groups, *n* = 6 samples/group (*n* = 6); **K** The relationship between the number of VM tubes and the intensity of FSLT3 or VE-Cadherin as detected by IHC. **L** IVIS in vivo imaging and the signal intensity quantitative results at study endpoint for the cecal orthotopic transplant tumor model of the HCT116 cell mixture with PCAF, *n* = 5. **M** Tumor growth curve of different group measured via IVIS in vivo imaging, *n* = 5mice/group. **N** Images of excised tumors in the intestine at study endpoint, n = 5/group. **O** Representative images of the liver at the study endpoint, including H&E staining showing liver metastases, Scale bar = 100 μm. **P** Quantification for number of tumors per mouse, abdominal metastases, and liver metastases, *n* = 5/group. **Q** Mice weight changes in each group during treatment, *n* = 5. **R** Representative images of the FAP and Masson’s staining in each group during treatment, *n* = 5, Scale bar =100 μm; **S** Quantification of the number of FAP positive cells and relative collagen content in three random fields for each sample in different groups, *n* = 5. All statistics are expressed as mean ± SD. *p < 0.05; **p < 0.01; ***p < 0.001; and ns, no significance. Statistical significance was calculated by one-way ANOVA with the Tukey post hoc test.

To evaluate the combinational anti-tumor effects of aFSTL3 and bevacizumab in vivo, we inoculated a mix of HCT116-Luc cancer cells with PCAF cells subcutaneously or in situ (Fig. 6A). In the subcutaneous model, both IVIS imaging and tumor growth curves showed that the combination of aFSTL3 and Bevacizumab significantly inhibited tumor growth and demonstrated superior tumor-suppressive effects compared to single-agent treatments (Fig. 6B, C). The combination also synergistically reduced the tumor weights and significantly prolonged survival (Fig. 6D–F). Moreover, TUNEL and Ki67 staining revealed that the combination therapy significantly increased apoptosis and inhibited tumor cell proliferation (Fig. 6G, H and Fig. S12**)**.

Interestingly, while Bevacizumab alone significantly suppressed angiogenesis, it did not obviously affect VM formation. In contrast, aFSTL3 treatment led to a marked reduction in VM formation, with modest inhibition of angiogenesis. The combination of aFSTL3 and Bevacizumab, however, demonstrated the most robust inhibition of both angiogenesis and VM formation, without inducing significant toxicity, as assessed by body weight monitoring, organ pathology, and biochemical analyses (Fig. 6I, J, Fig. S13 and Table S12, 13). Additionally, the combination treatment significantly suppressed AKT activation and downregulated VE-Cadherin expression in tumors. The number of VM formations was strongly positively correlated with FSTL3, pAKT, and VE-Cadherin staining, further underscoring the critical role of the FSTL3/pAKT/VE-Cadherin axis in regulating VM formation (Fig. 6K and Fig. S14**)**.

In the orthotopic model, both aFSTL3 and Bevacizumab effectively suppressed tumor growth, with the combination treatment demonstrating the highest anti-tumor efficacy and some mice achieved complete tumor remission (Fig. 6L-M). Notably, gross tumor specimens from the combination group showed a significant reduction in abdominal metastases and a marked decrease in liver metastasis (Fig. 6N–P). These findings suggest that targeting FSTL3 with aFSTL3 in combination with Bevacizumab significantly enhances the pre-clinical therapeutic efficacy of Bevacizumab without affecting body weight (Fig. 6Q). In addition, aFSTL3 significantly reduced the number of CAFs and collagen content (Fig. 6R, S). Therefore, aFSTL3 combined with Bevacizumab holds promise as a potent therapeutic strategy to improve the prognosis of colon cancer.

## Discussion

VM represents an alternative vascularization mechanism independent of endothelial cells, contributing significantly to therapy resistance, tumor progression, and metastasis [7]. In this study, we identify FSTL3 as a key factor predominantly expressed by CCAFs, which plays a pivotal role in VM formation and correlates with poor prognosis in colon cancer patients. These findings position FSTL3 as a promising therapeutic target for colon cancer.

VM has emerged as a prognostic marker, with its presence associated with worse outcomes in various malignancies, including colon cancer [23–25, 86]. Despite its clinical relevance, the mechanisms driving VM formation remain poorly understood, and VM has yet to be effectively targeted in clinical practice. Traditional anti-angiogenic therapies, such as bevacizumab, fail to address this alternative vascularization pathway [87, 88]. Our study not only identifies FSTL3 as a novel target capable of disrupting VM but also highlights the potential of combining anti-FSTL3 therapy with bevacizumab. This dual-target strategy could synergistically inhibit both VM and angiogenesis, offering a comprehensive approach to overcoming anti-angiogenic resistance and improving therapeutic outcomes.

CCAFs are integral components of the TME, mediating tumor progression, ECM remodeling, and immune suppression in colon cancer [26, 31, 33]. However, their therapeutic targeting has been challenging due to heterogeneity and a lack of specificity. Our findings reveal that FSTL3^+^ CCAFs represent a distinct subpopulation that drives VM formation through the AKT/mTOR/VE-Cadherin signaling axis, thereby enhancing cancer cell aggressiveness. Targeting FSTL3 offers a dual-action therapeutic approach by simultaneously suppressing VM and CCAF-mediated tumor support. While previous studies have focused on FSTL3’s tumor-intrinsic roles [40, 43, 74], our data highlight its dual function as a therapeutic target capable of inhibiting both CCAFs and tumor cells.

Our study is the first to employ single-cell RNA sequencing (scRNA-seq) to investigate TME differences between well-defined VM and non-VM tissues in human colon cancer samples. Despite limitations in sample size, our scRNA-seq analysis revealed that non-VM/FSTL3-low patients exhibited enrichment in T cells, B cells, and plasma B cells, whereas macrophages and CCAFs—cell types associated with an immunosuppressive TME—were enriched in VM/FSTL3-high patients. This observation aligns with previous reports [39, 76] and suggests that FSTL3 may play a critical role in shaping the TME. Future studies should explore whether FSTL3 modulates the TME and whether targeting FSTL3 could enhance the efficacy of immunotherapy, particularly in mismatch repair-proficient (pMMR) colon cancer patients, who typically exhibit poor responses to current immunotherapies [89].

Traditional VEGF-targeting therapies induce hypoxia within the TME, paradoxically upregulating VEGF expression and promoting resistance through compensatory mechanisms [87]. Our findings reveal an alternative, VEGF-independent, hypoxia-responsive pathway mediated by FSTL3, which supports blood and nutrient supply via VM rather than endothelial cell-driven angiogenesis. We identify TfR1 as a critical interactor of FSTL3 on cancer cells, activating the AKT/mTOR/VE-Cadherin signaling axis to promote VM formation. This interaction underscores an innovative mechanism of tumor vascularization with significant therapeutic implications. However, whether TfR1 serves as FSTL3’s receptor on endothelial cells remains unclear and warrants further investigation.

While anti-VEGF therapies are effective in managing tumor vascularization, their use is often limited by side effects such as hypertension, thromboembolism, gastrointestinal perforation, and impaired wound healing, necessitating treatment holidays. Unfortunately, these breaks in treatment can lead to rapid vascular regrowth and tumor progression [90–92]. Anti-FSTL3 therapy offers a viable alternative during these critical periods. Unlike VEGF inhibitors, FSTL3-targeting antibodies specifically inhibit VM without significantly affecting endothelial cell-mediated angiogenesis, making them potentially suitable for use during treatment breaks when traditional anti-angiogenic agents are contraindicated. This approach provides a complementary strategy to maintain tumor suppression, addressing a critical gap in managing therapy resistance and tumor recurrence.

## Conclusions

In summary, our study highlights the dual significance of FSTL3 in VM formation and CCAF function. By targeting the FSTL3/TfR1 axis, we propose a novel therapeutic strategy that may overcome the limitations of current anti-angiogenic and stromal-targeted therapies. The ability to simultaneously inhibit VM, angiogenesis, and CAF-mediated tumor support represents a promising advance in colon cancer treatment. Future clinical trials validating FSTL3-targeted therapies could pave the way for more effective combination treatments, ultimately improving patient outcomes and establishing FSTL3 as a cornerstone in the development of anti-tumor strategies.

## Supplementary information

Supplementary Figures

Supplementary Table

Uncropped gels for Western Blots

## Data Availability

The RNA-seq data have been uploaded to the GEO database at accession number GSE253699. The raw scRNA-seq data reported in this paper have been deposited in the Genome Sequence Archive (Genomics, Proteomics & Bioinformatics 2021) in National Genomics Data Center (Nucleic Acids Res 2022), China National Center for Bioinformation / Beijing Institute of Genomics, Chinese Academy of Sciences (GSA-Human: HRA009387) that are publicly accessible at https://ngdc.cncb.ac.cn/gsa-human [93, 94]. The datasets generated during the current study were available in TCGA and GEO databases, including TCGA-COAD, GSE39582, GSE17536, GSE166555, and GSE240789 cohorts. All other raw data are available upon request from the corresponding author.
