# Supplementary material for: Cancer-associated fibroblasts expressing FSTL3 promote vasculogenic mimicry formation and drive colon cancer malignancy
Source: Cell Death Dis. 2025 Oct 6;16(1):706. doi: 10.1038/s41419-025-08009-w (PMC12501291; doi:10.1038/s41419-025-08009-w)
Supplement: Supplementary file 1 — Supplementary Figures [file 41419_2025_8009_MOESM1_ESM.docx]

**Cancer-Associated Fibroblasts Expressing FSTL3 Promote Vasculogenic Mimicry Formation and Drive Colon Cancer Malignancy**

Leqian Ying^1#^, Yini Zhu^2#^, Lu Zhang^1^, Min Ji^1^, Meidan Wang^2,3^, Lei Dong^1^, Zhengcheng Yun^1^, Yanping Chen^1^, Jingyi Zhou^1^, Chunchun Huang^1^, Shengwei Zhang^4^, Xuhong Yang^5^, Hui Yang^6^, Guichun Huang^1*^, Shukui Qin^7*^, Jinbing Xie^5*^ and Lin Liu^1*^

^1^Department of Oncology, Zhongda Hospital, Medical School, Southeast University, Nanjing 210009, Jiangsu, China.

^2^Department of Microbiology and Immunology, Medical School of Southeast University, Nanjing 210009, Jiangsu, China.

^3^Department of Radiation Oncology, University of Freiburg Faculty of Medicine, Freiburg 79106, Germany.

^4^College of plant protection, Yangzhou university, Yangzhou, 225100, Jiangsu, China.

^5^Nurturing Center of Jiangsu Province for State Laboratory of AI Imaging & Interventional Radiology; Basic Medicine Research and Innovation Center of Ministry of Education; State Key Laboratory of Digital Medical Engineering; Department of Radiology, Zhongda Hospital, Medical School of Southeast University, Nanjing 210009, Jiangsu, China.

^6^Department of Biochemistry and Molecular Biology, Medical School of Southeast University, Nanjing 210009, Jiangsu, China.

^7^GI Cancer Center, Nanjing Tianyinshan Hospital, China Pharmaceutical University, Nanjing 211100, Jiangsu, China.

**Supplementary Figures**


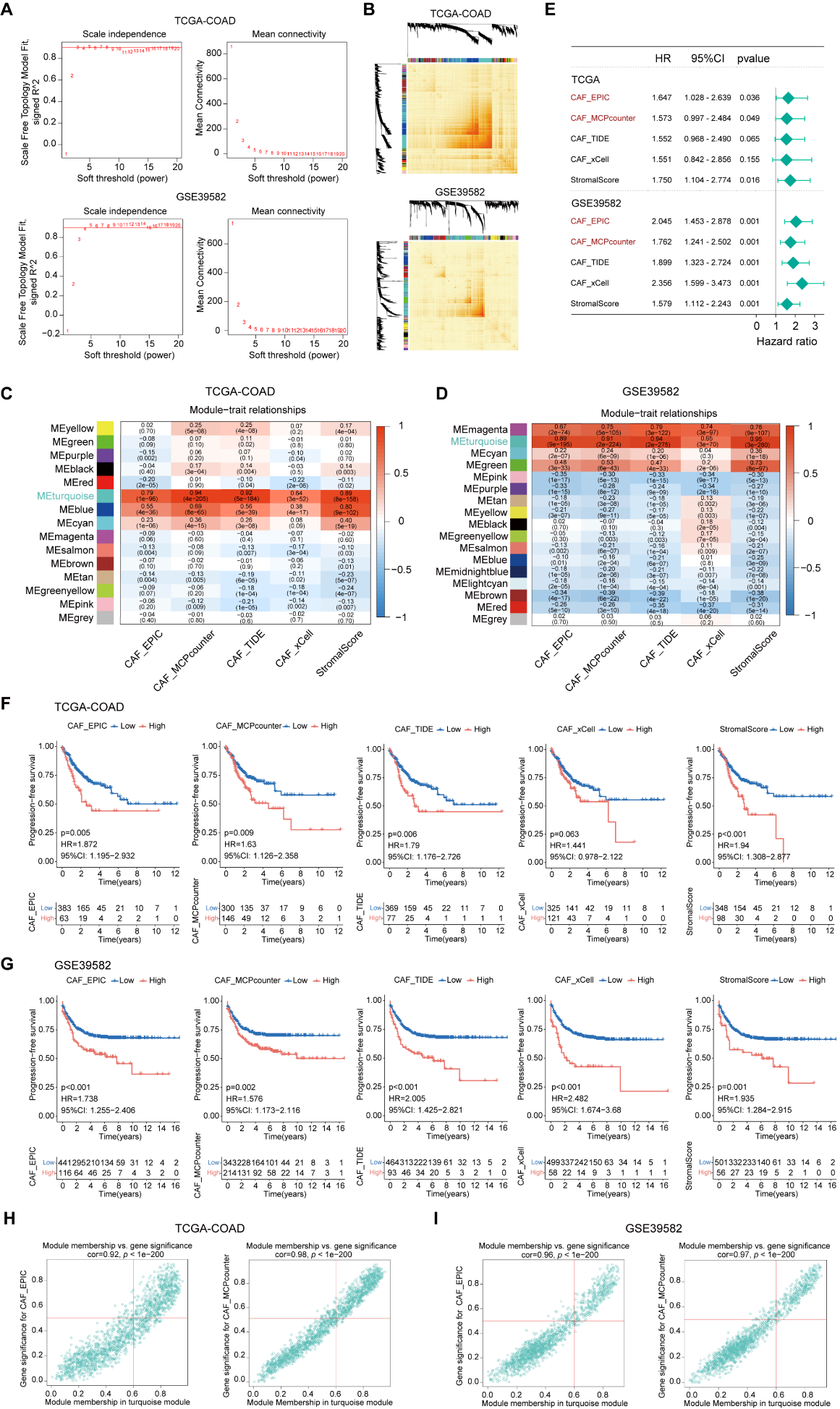


**Fig. S1: Screening of key genes related to CCAF-related VM in colon cancer.**

**A,** Soft-thresholding powers (β) were selected when the model R squared approached 1 in the TCGA-COAD and GSE39582 cohorts. **B**, Co-expression gene modules with TOMplot in TCGA_COAD and GSE39582 cohorts. **C, D**, Heat map revealing the correlations between each gene module eigengene and the phenotype of CCAF infiltration in TCGA-COAD (**C**) and GSE39582 (**D**) cohorts. **E**, The forest plot showing the Univariate Cox regression analysis of five CCAF infiltration enrichment scores. **F**, **G**, Kaplan–Meier analysis showing PFS was generally shorter in the high CAF infiltration group, as determined by algorithms including CAF_EPIC, CAF_MCPcount, CAF_TIDE, CAF_xCell, and Stromalscore, compared to the low infiltration group in patients from the TCGA-COAD (**F**) and GSE39582 (**G**) cohorts. **H**, **I**, Scatter plots visualizing the Module Membership (MM) and Gene Significance (GS) of genes within the turquoise module. All statistics are expressed as mean ± SD. Pearson correlation analysis was applied for the relationship between two indicators in (C) and (D). Statistical analysis of survival time was performed using log-rank tests in (F) and (G).


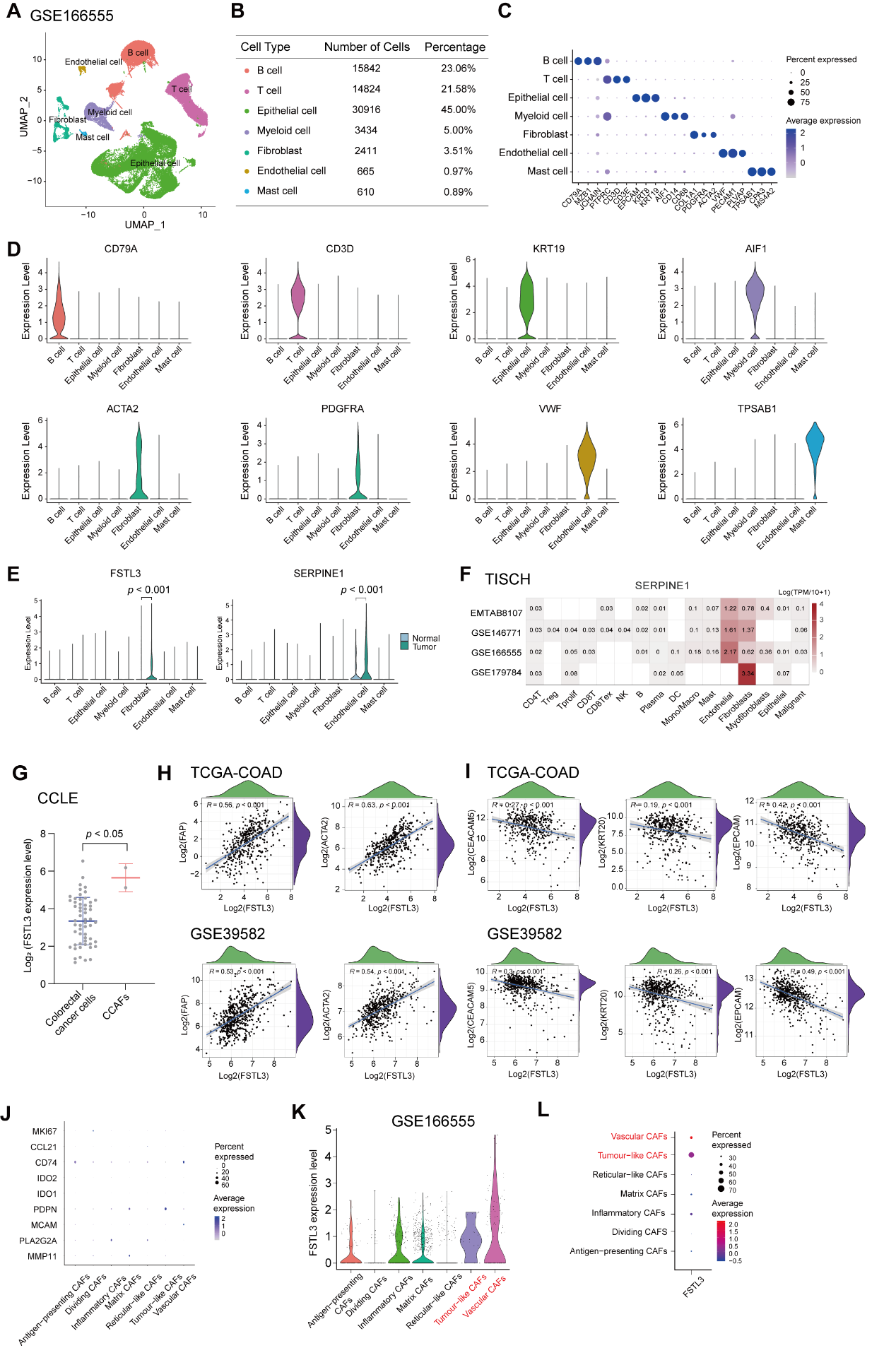


**Fig. S2: Performing expression levels on scRNA-seq and RNA-seq of colon cancer cohorts.**

**A**, Cells of the GSE166555 cohort were clustered into seven subtypes using the UMAP dimensionality reduction algorithm. **B**, Number and proportions of the seven subtypes. **C**, Expression levels of the labelled genes in seven subtypes. **D**, Violin plot showed the expression levels of labelled genes in different cell subtypes. **E**, Violin plot showed the expression levels of FSTL3 and SERPINE1 in normal and tumor tissues. **F**, The heatmap displayed the expression of SERPINE1 in four different scRNA-seq cohorts in the TISCH online database. **G**, The mRNA expression of FSTL3 in colorectal cancer cells (n=56) and CCAFs (n=2) based on the CCLE database. **H**, Correlation analysis graph of FSTL3 with mRNA expression levels of FAP and ACTA2 in TCGA-COAD and GSE39582 cohorts. **I**, Correlation analysis graph of FSTL3 with mRNA expression levels of epithelial markers (CEACAM5, KRT20, EPCAM) in TCGA-COAD and GSE39582 cohorts. **J**, CCAFs subtype was also divided into seven subgroups according to the expression levels of the labelled genes. **K, L**, Violin plot and proportion distribution of FSTL3 expression levels in the CAFs subpopulation of the GSE166555 cohort. All statistics are expressed as mean ± SD. Statistical significance was calculated by two-tailed t test in (E) and (G). Pearson correlation analysis was applied for the relationship between two indicators in (H) and (I).


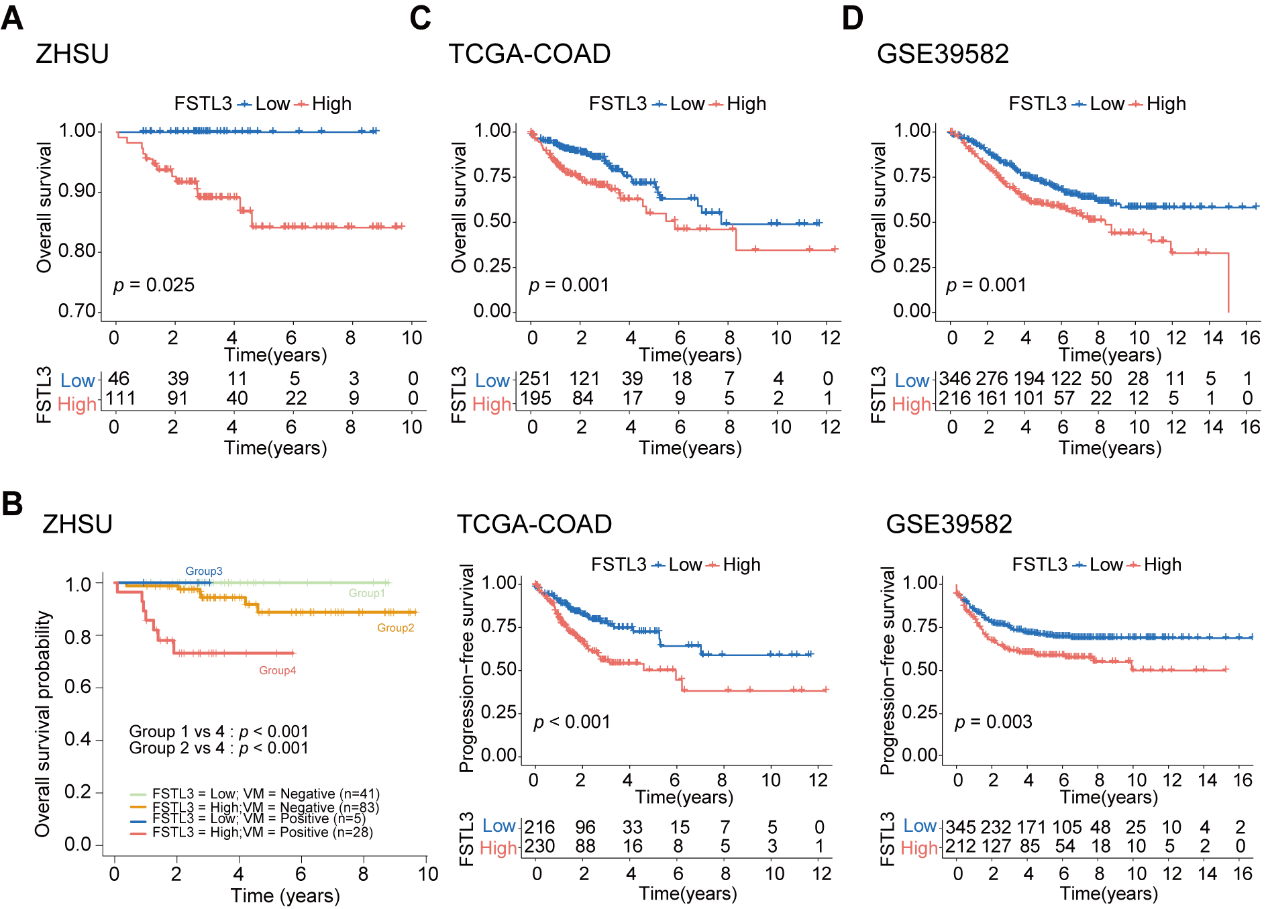


**Fig. S3: Kaplan-Meier survival curves in the three cohorts.**

**A,** Kaplan-Meier survival curves on OS and PFS of the ZHSU cohort stratified by low and high FSTL3 expression detected by IHC. **B**, Kaplan-Meier survival curves on OS of the ZHSU cohort stratified by FSTL3 expression and VM status. **C, D**, Kaplan-Meier survival curves on OS and PFS of TCGA-COAD and GSE39582 cohorts stratified by low and high FSTL3 mRNA expression. All statistics are expressed as mean ± SD. Statistical analysis of survival time was performed using log-rank tests in (A), (C) and (D), and Gehan-Breslow-Wilcoxon test in (B).


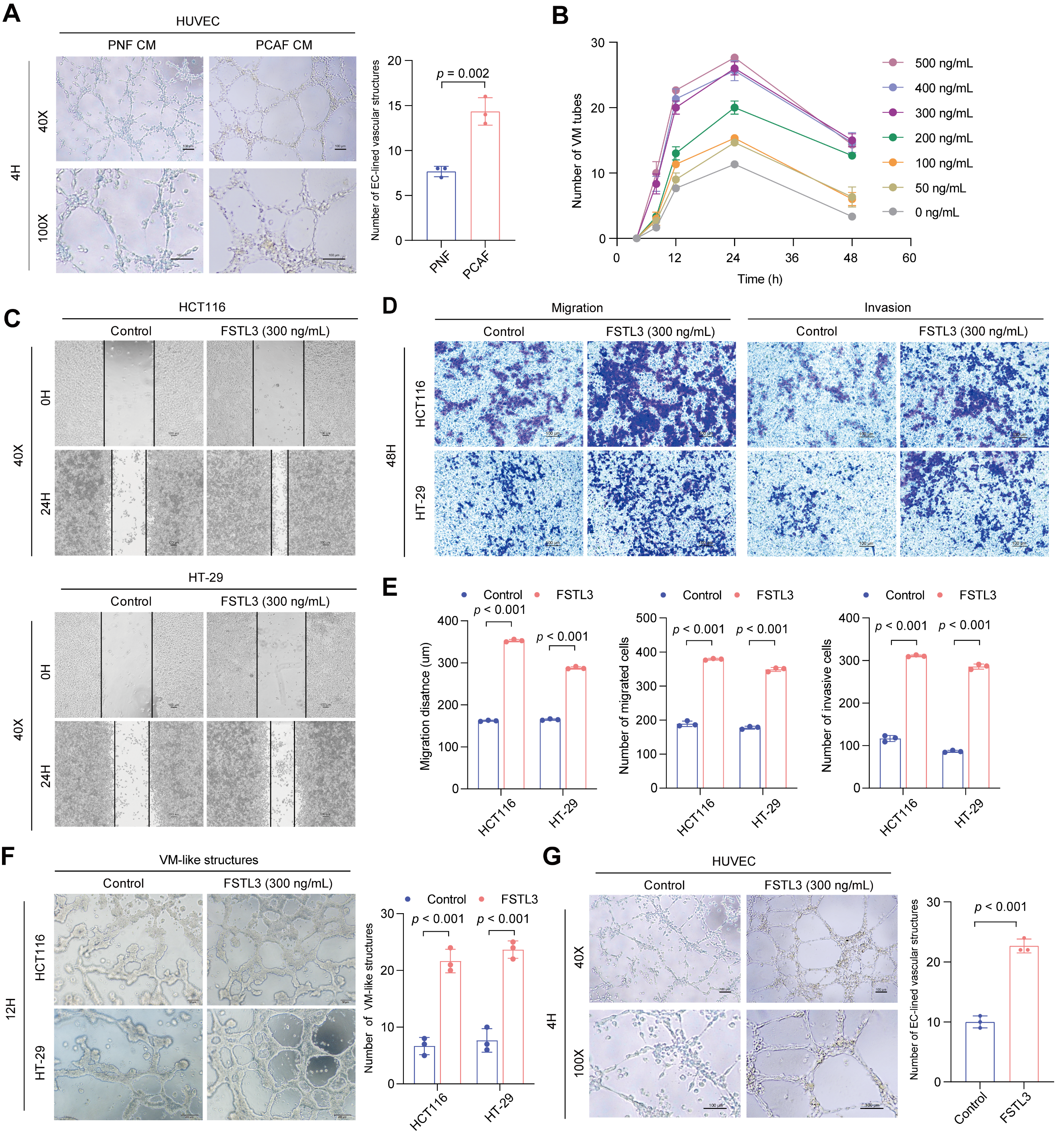


**Fig. S4: FSTL3 promoted VM formation and enhanced invasion.**

**A,** The CM derived from PCAF promoted vessel-like structure formation of HUVEC compared with the PNF CM. Scale bar =100 um, n=3; **B,** The line graph showed the time and concentration of FSTL3 in promoting tube-like structure formation in HCT116 cells. (n = 3, biologically independent samples); **C,** Scratch experiment of FSTL3 cells treated with HCT116 and HT-29 cells. Scale bar =100 um, n=3; **D,** Transwell experiment of FSTL3 treated with HCT116 and HT-29. Scale bar =100 um, n=3; **E,** The quantification of scratch and transwell experiments about **C** and **D**; **F**. Tube-like structure formation of HCT116 and HT-29 cells treated with FSTL3 for 12h and its quantification, Scale bar =100 um, n=3; **G,** Vessel-like structure formation of HUVEC treated with FSTL3 for 4h and its quantification, Scale bar =100 um, n=3. All statistics are expressed as mean ± SD. Statistical significance was calculated by two-tailed t test.


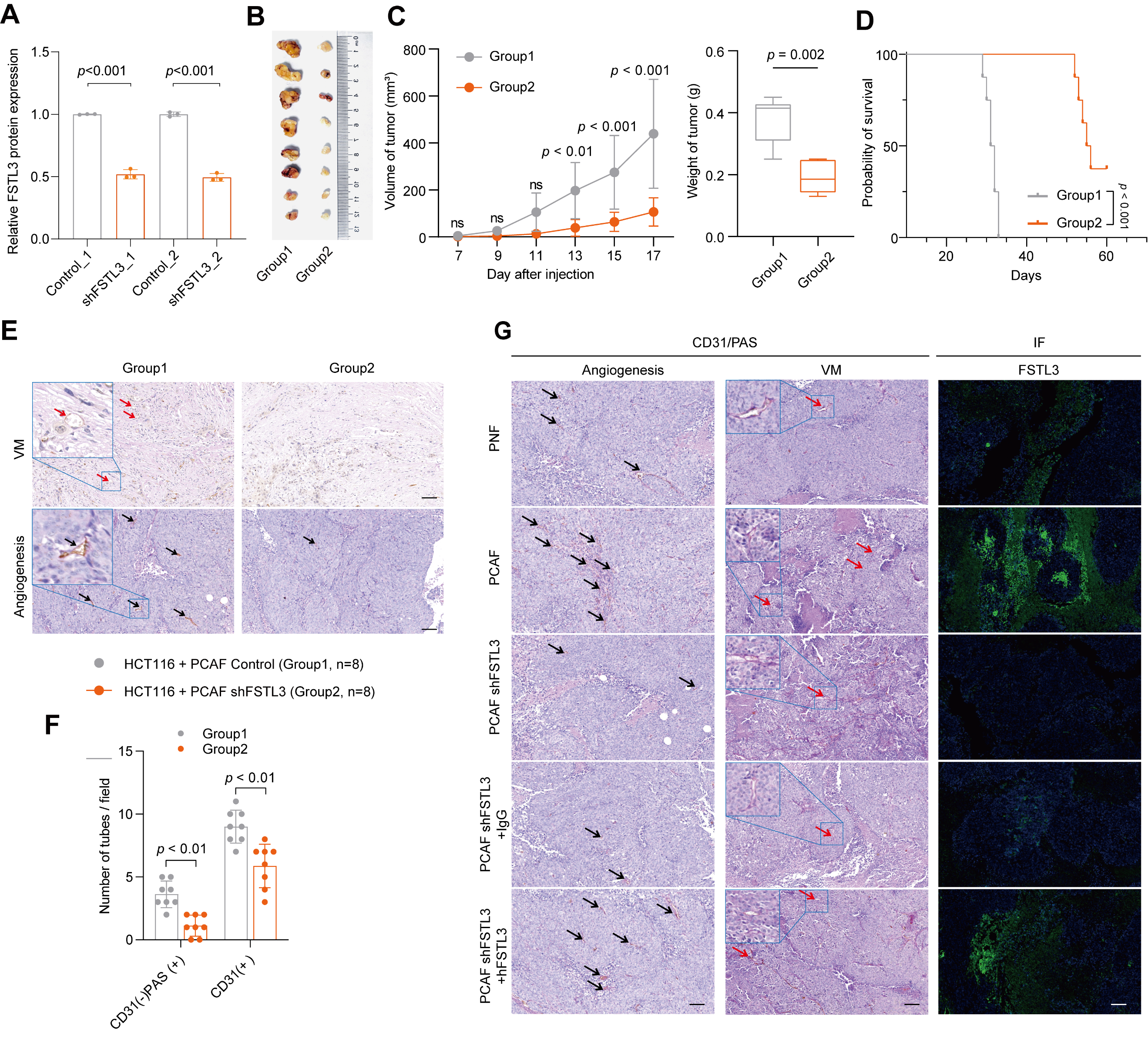


**Fig. S5: In vivo validation of FSTL3 knockdown inhibiting VM and colon cancer malignancy.**

**A,** The quantification of FSTL3 in the intracellular protein of PCAF with shFSTL3. n=3; **B,** Images of xenograft tumors, n = 8/group; **C,** Line graph depicting changes in tumor volume; Bar graph comparing tumor weights, n = 8/group; **D,** Survival curve, n = 8/group; **E**, CD31 and PAS IHC staining for the tissue. The black arrow represents VM (-) which is CD31^+^PAS^-^, and the red arrow represents VM (+) which is CD31^-^PAS^+^ (Scale bar =100 um, n=8); **F,** Number of VM tubes and blood vessels, n = 8/group; **G**, CD31/PAS IHC and FSTL3 IF staining for the tissue. The black arrow represents VM (-) which is CD31^+^PAS^-^, and the red arrow represents VM (+) which is CD31^-^PAS^+^ (Scale bar =100 um, n=5); All statistics are expressed as mean ± SD. Statistical significance was calculated by two-tailed t test. The Kaplan-Meier method with the Log-rank test was used to compare survival time between two groups.


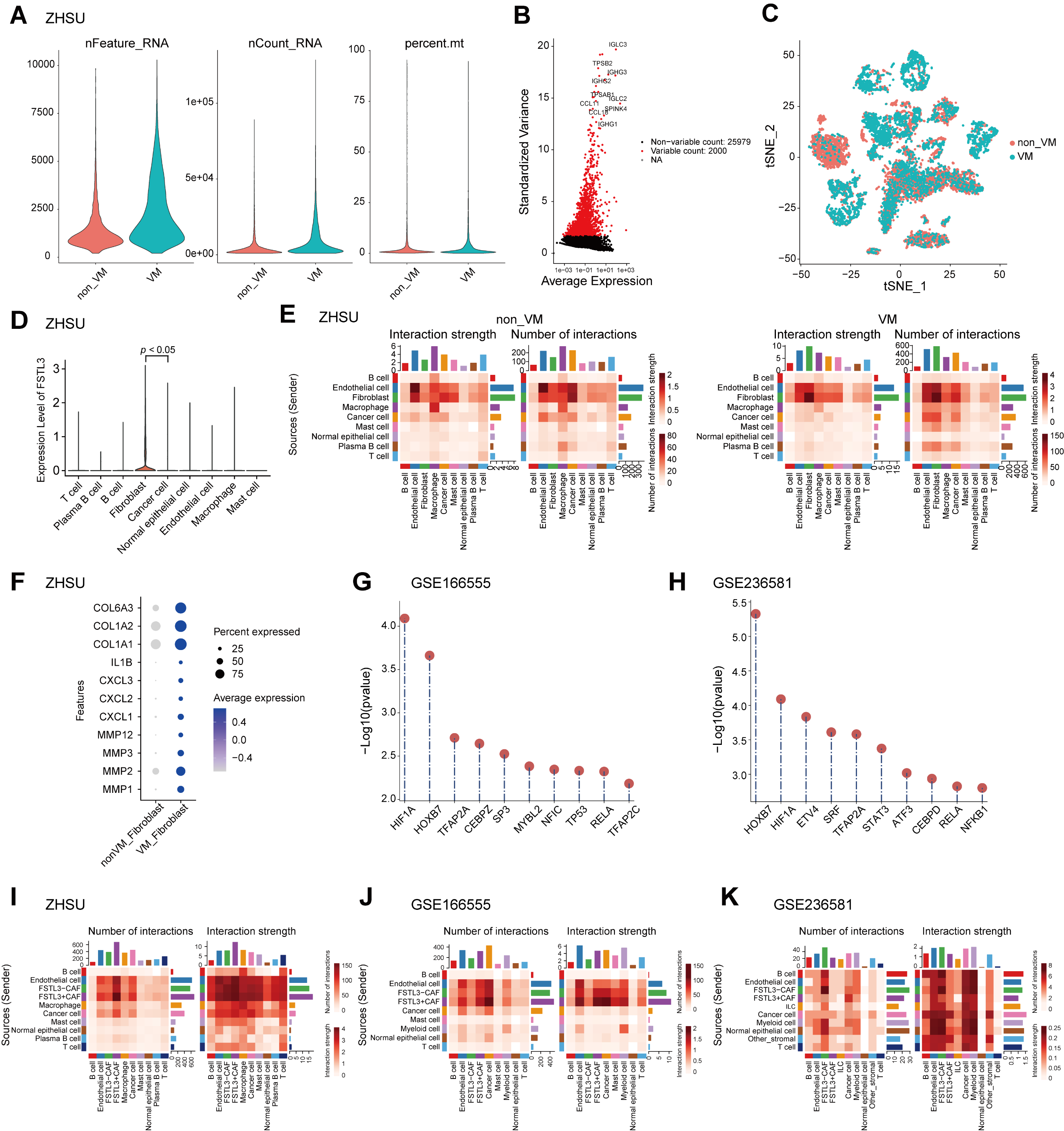


**Fig. S6: Performing cell subtyping on scRNA-seq of non_VM and VM patients according to marker genes.**

**A,** The quality control summary of scRNA-seq data was shown in a violin plot. **B,** Scatter plots showed the distribution of hypervariable genes, and the first ten hypervariable genes were labelled. **C,** The tSNE plot after applying the harmony function. **D,** Violin plot showed the expression levels of FSTL3 in nine subtypes. **E**, The heatmaps showed the number and strength of interactions between nine subtypes in ZHSU cohort, based on CellChat analysis. **F**, Bubble plot showed the ECM-related marker genes in non_VM and VM CCAFs; **G, H**, TRRUST Transcription Factor Enrichment analysis using the top 250 genes upregulated in FSTL3^+^ CCAF in GSE166555 and GSE 236581 cohorts, ranked by P-value, with a log_2_ fold change greater than 0.25; **I-K**, The heatmap showed the number and strength of interactions between FSTL3^+^ CCAFs and other subtypes in ZHSU, GSE166555, and GSE 236581 cohorts, based on CellChat analysis. All statistics are expressed as mean ± SD. Statistical significance was calculated by two-tailed t test.


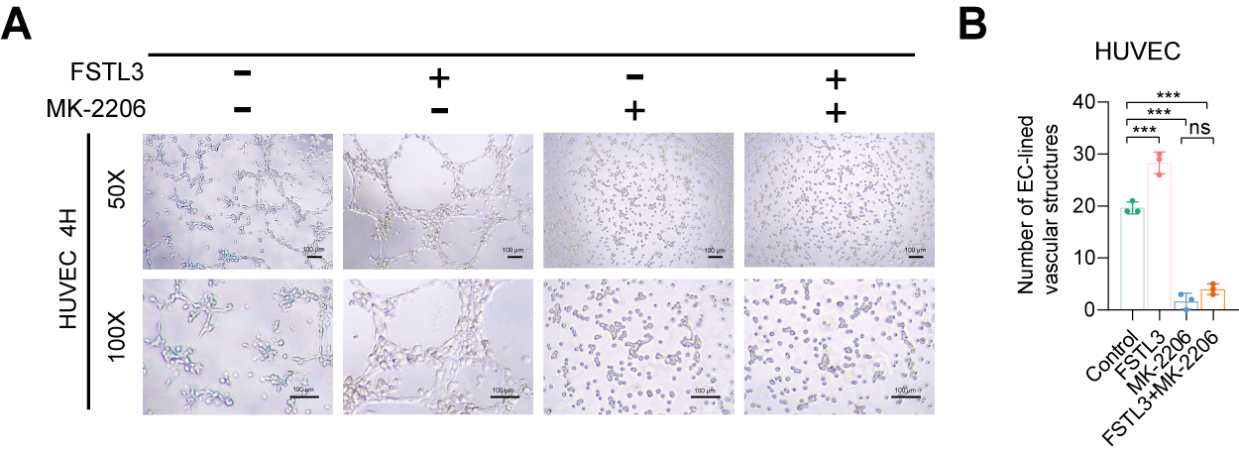


**Fig. S7: FSTL3 induces vessel-like structure formation in HUVECs.**

**A,** Representative images showing vessel-like structure formation in HUVECs under different treatments, Scale bar =100 um; **B**, Quantification of tube formation in HUVECs under different treatments, n = 3; All statistics are expressed as mean ± SD. * *p* < 0.05, ** *p* < 0.01, *** *p* < 0.001 and ns, no significance. Statistical significance was calculated by one-way ANOVA with the Tukey post hoc test.


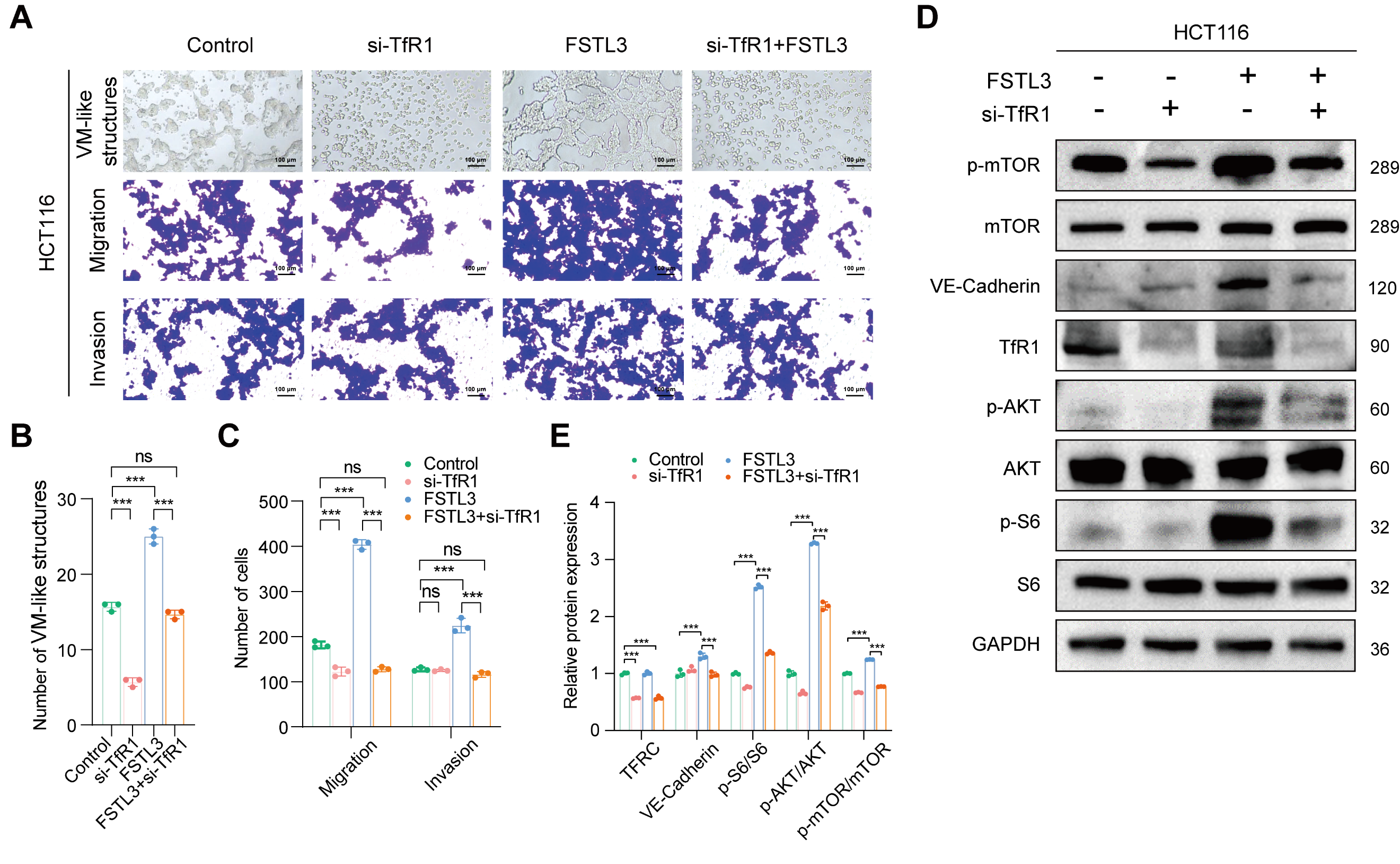


**Fig. S8: The ability of FSTL3 to induce migration, invasion, and vessel-like structure formation in si-TfR1 HCT116 cells is diminished.**

**A,** Representative images of tube formation, cell migration, and cell invasion in vitro; Scale bar =100 um, n = 3; **B-C,** Quantification of tube formation (**B**) and migration or invasion ability of HCT116 cells (**C**), n = 3; **D-E,** Western blot images (**D**) and quantification (**E**) illustrating protein expression levels in the TfR1/AKT/mTOR pathways in si-TfR1 HCT116 cells; All statistics are expressed as mean ± SD. *, *p* < 0.05; **, *p* < 0.01; ***, *p* < 0.001; and ns, no significance. Statistical significance was calculated by one-way ANOVA with the Tukey post hoc test.


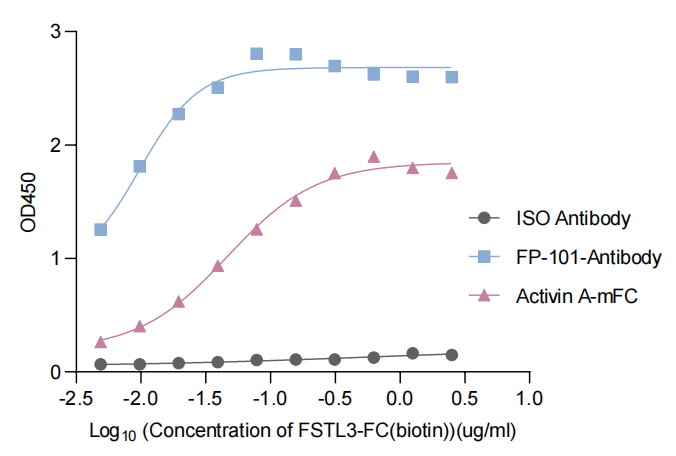


**Fig. S9: The affinity of FP101 antibody.**

FSTL3-FC (biotin) was bonded to ISO antibody, FP-101 antibody (aFSTL3), and Activin A by ELISA. Activin A was used as a positive control.


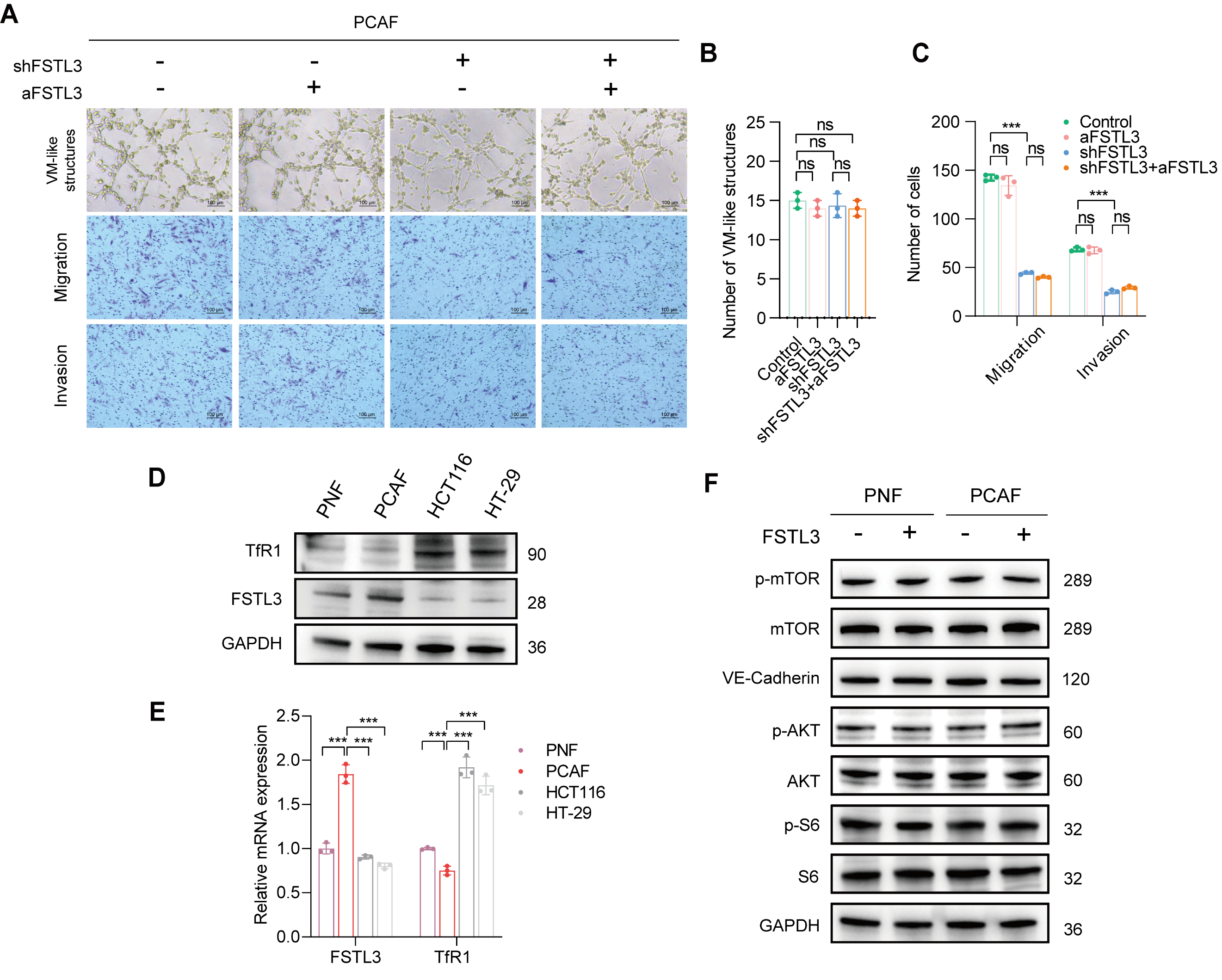


**Fig. S10: The effect of aFSTL3 on migration, invasion, and vessel-like structure formation.**

**A,** Representative images of PCAF tube formation, migration, and invasion in vitro; Scale bar =100 um, n = 3; B-**C,** Quantification of tube formation **(B)** and migration or invasion ability of PCAF **(C)**, n = 3; **D-E,** Western blot and qPCR analysis of FSTL3 and TfR1 expression in four cell types; **F,** Western blot images illustrating protein expression levels in the AKT/mTOR pathways in PNF and PCAF; All statistics are expressed as mean ± SD. *, p < 0.05; **, p < 0.01; ***, p < 0.001; and ns, no significance. Statistical significance was calculated by one-way ANOVA with the Tukey post hoc test.


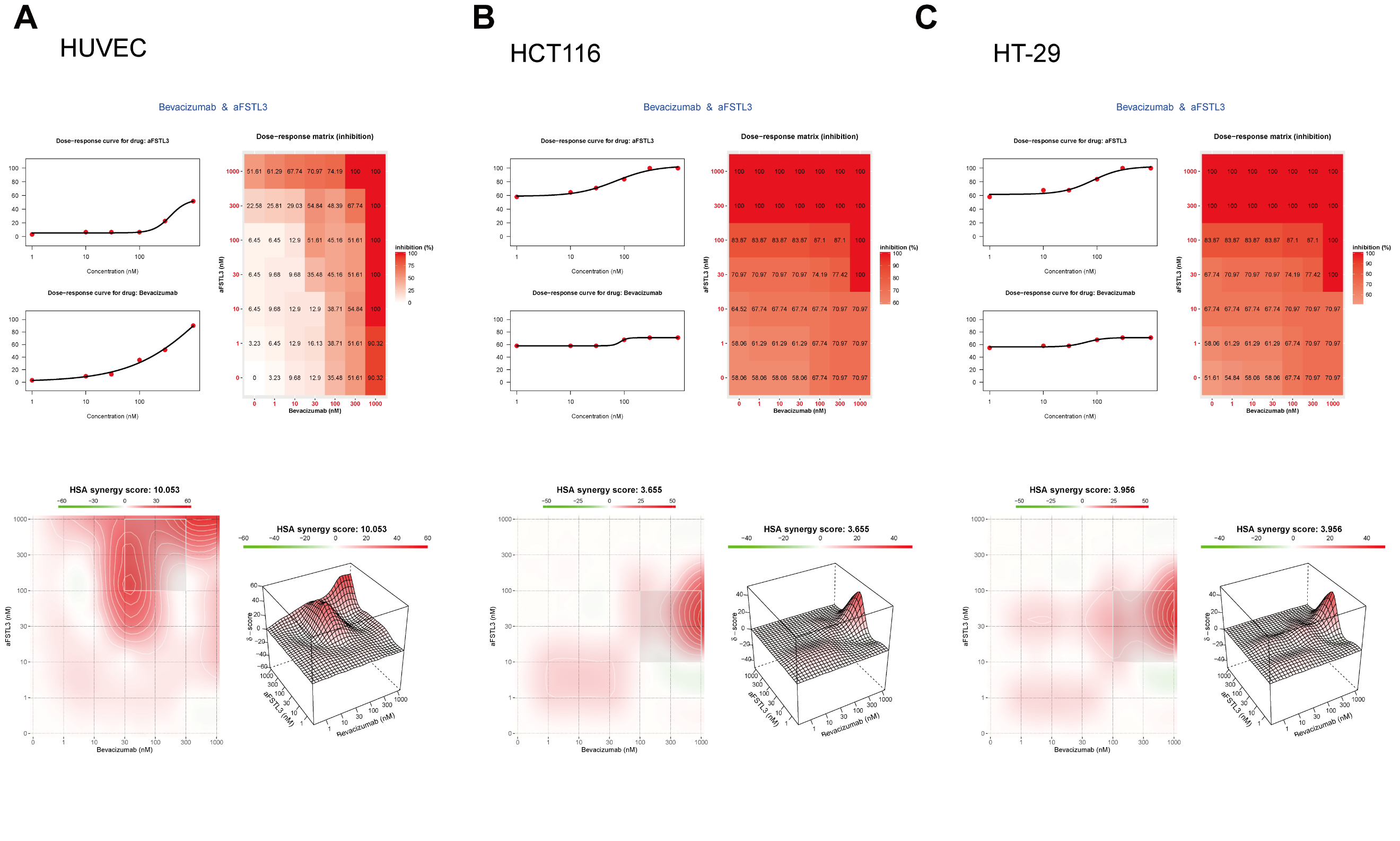


**Fig. S11: Analysis of the synergistic effect of aFSTL3 and bevacizumab.**

**A-C,** The HAS synergistic scores of drug combinations targeting tube formation.


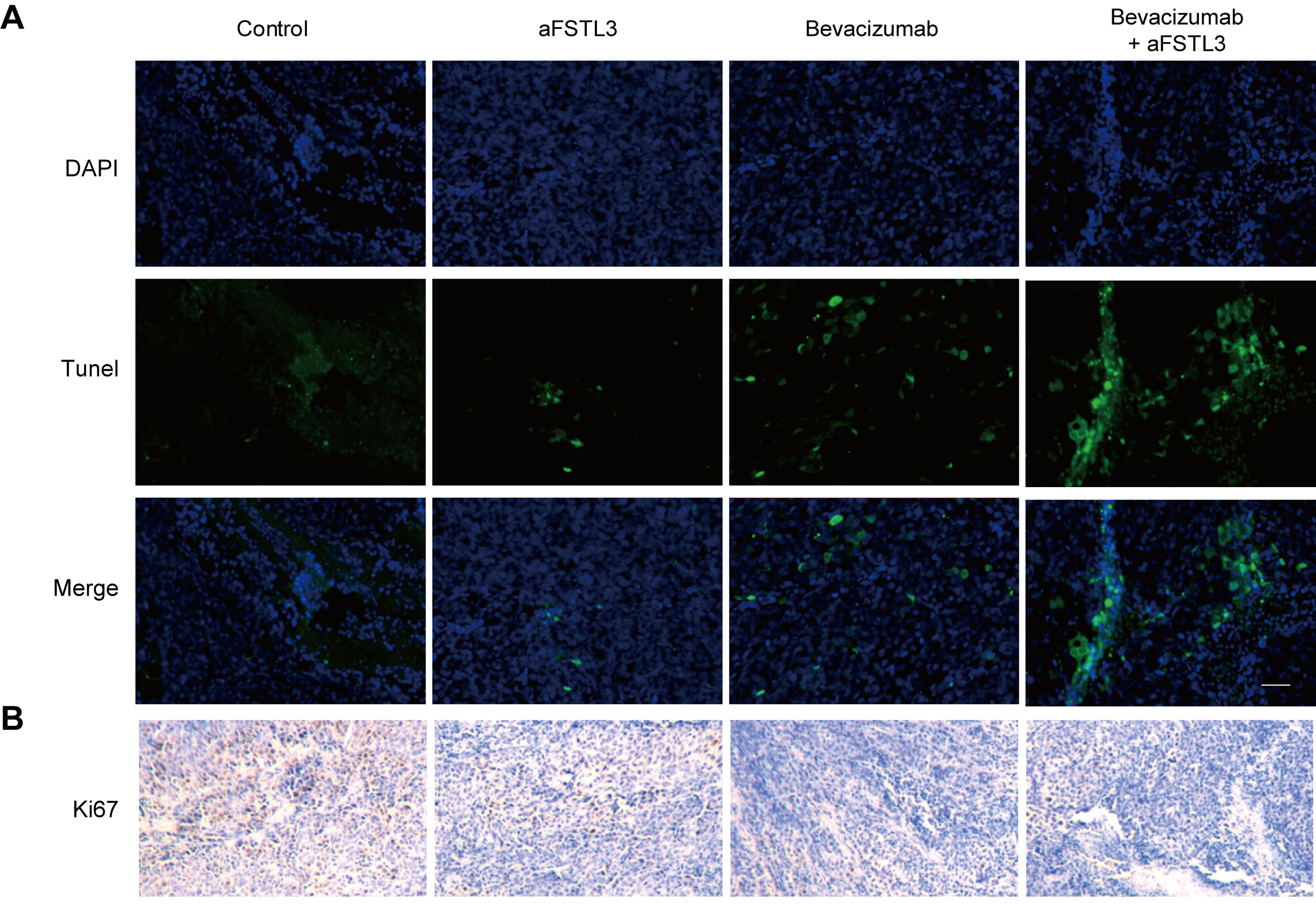


**Fig. S12: Tunel fluorescence and Ki67 staining.**

**A, B,** The Tunel fluorescence and Ki67 staining of subcutaneous xenograft tumors. Scale bar =100 um. (n = 6, biologically independent samples).


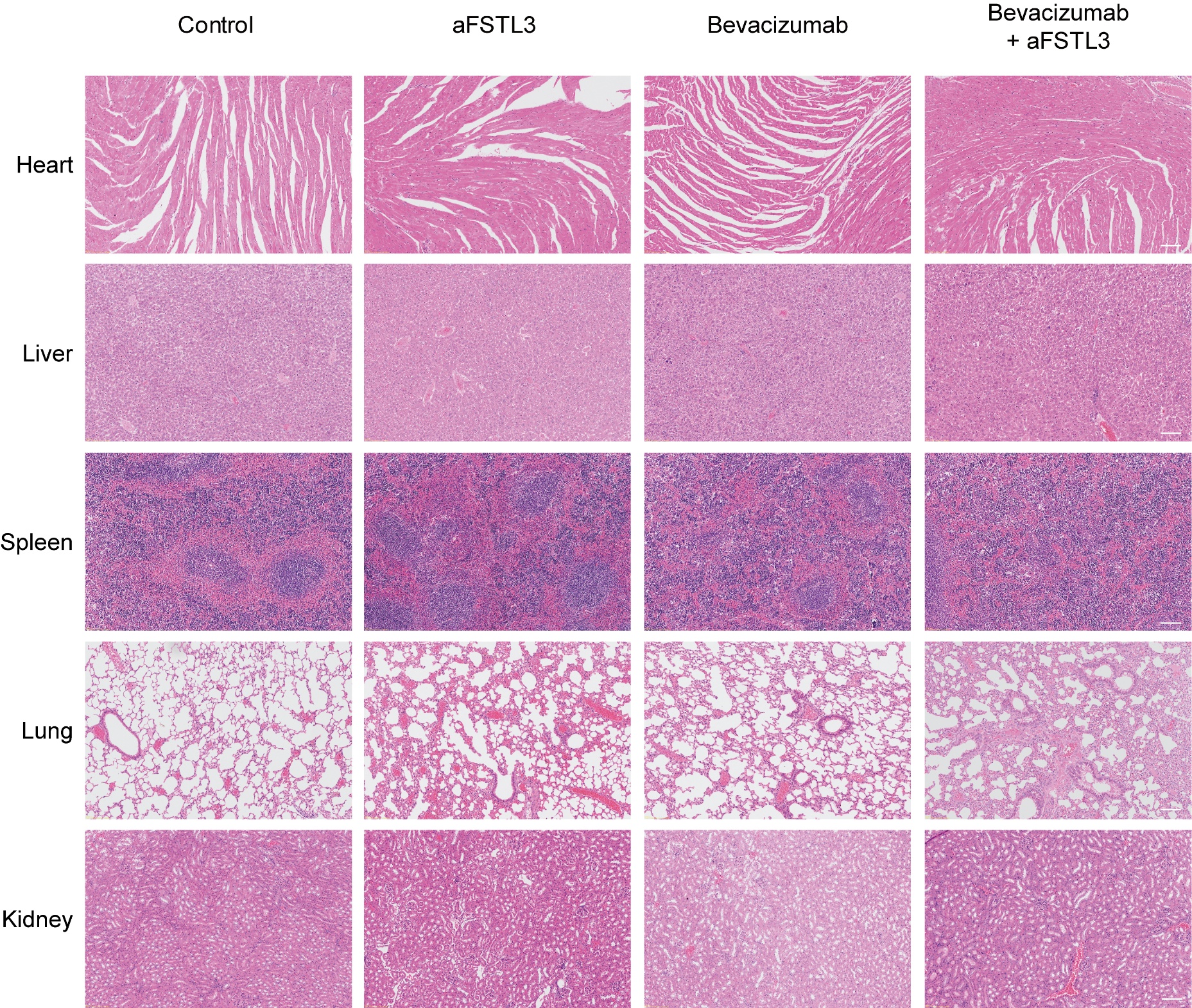


**Fig. S13: HE staining of heart, liver, spleen, lung and kidney tissue sections is performed after treatments.**

Seven days after injection of various antibodies, mice were euthanized and viscera (heart, liver, spleen, lungs, and kidneys) were excised, followed by HE staining to detect potential damage. Scale bar=50µm. (n = 3, biologically independent samples).


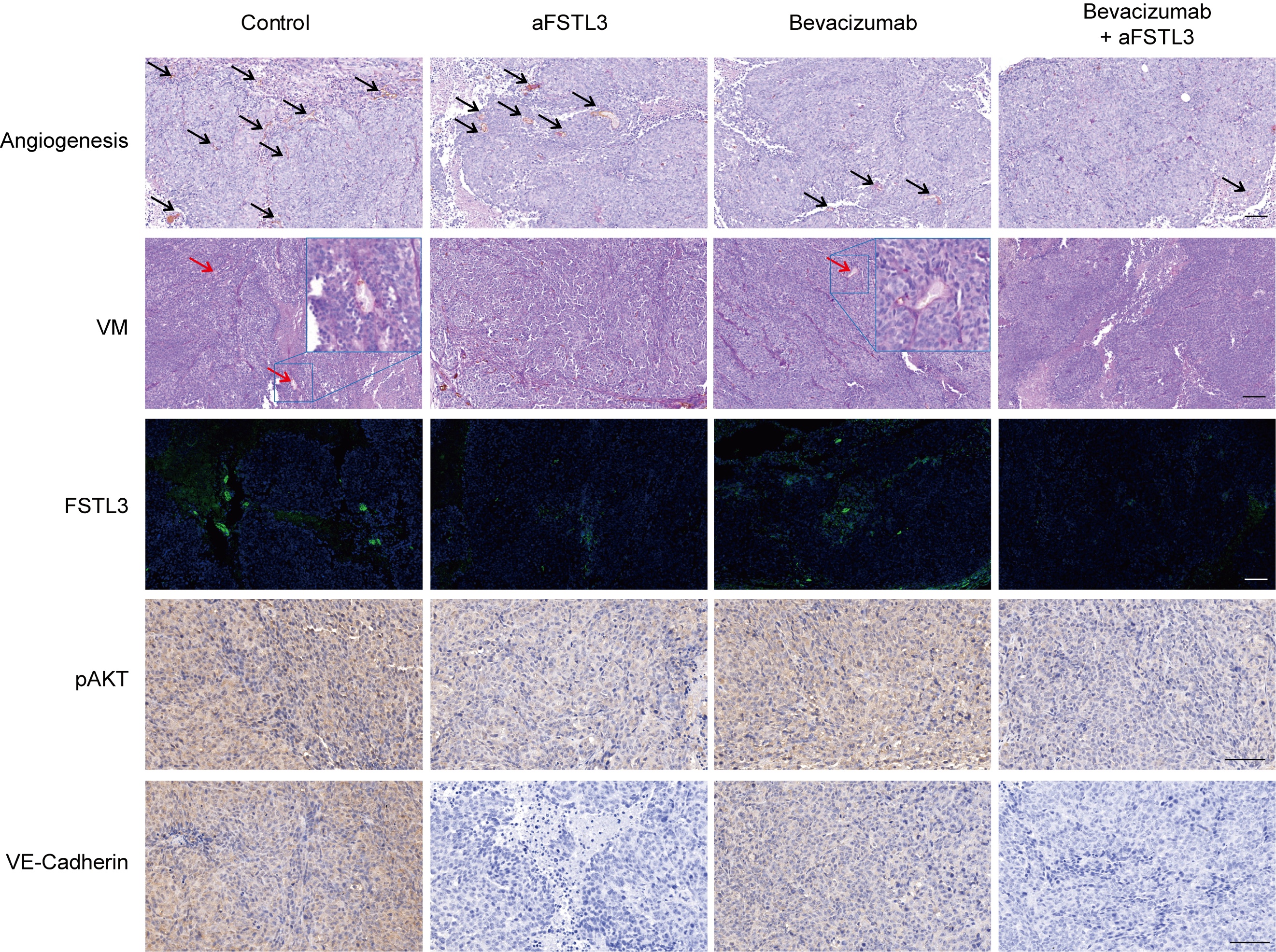


**Fig. S14: FSTL3 fluorescence and IHC staining.**

FSTL3 fluorescence and IHC staining of CD31/PAS, pAKT, and VE-Cadherin of subcutaneous xenograft tumors. Scale bar =100 um. (n = 6, biologically independent samples).
