# Supplementary material for: Cancer-associated fibroblasts expressing FSTL3 promote vasculogenic mimicry formation and drive colon cancer malignancy
Source: Cell Death Dis. 2025 Oct 6;16(1):706. doi: 10.1038/s41419-025-08009-w (PMC12501291; doi:10.1038/s41419-025-08009-w)

Uncropped gels for Western Blots

Uncropped gels for Western Blots in Figure 2F

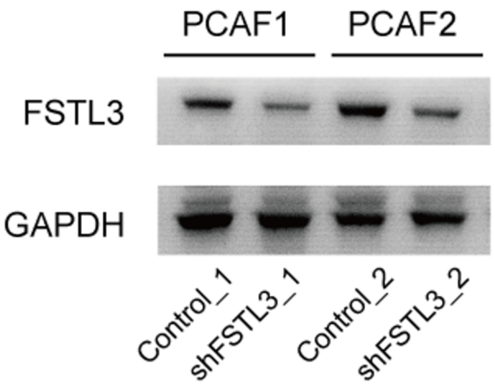

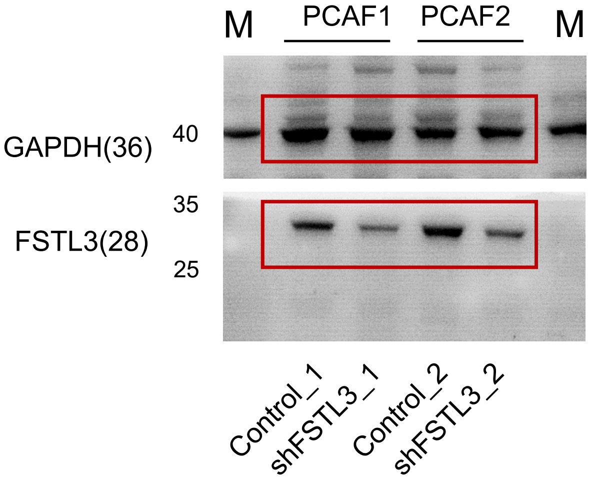

Uncropped gels for Western Blots in Figure 4H

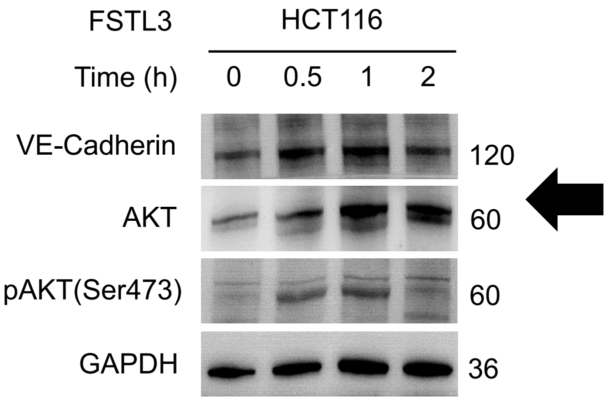

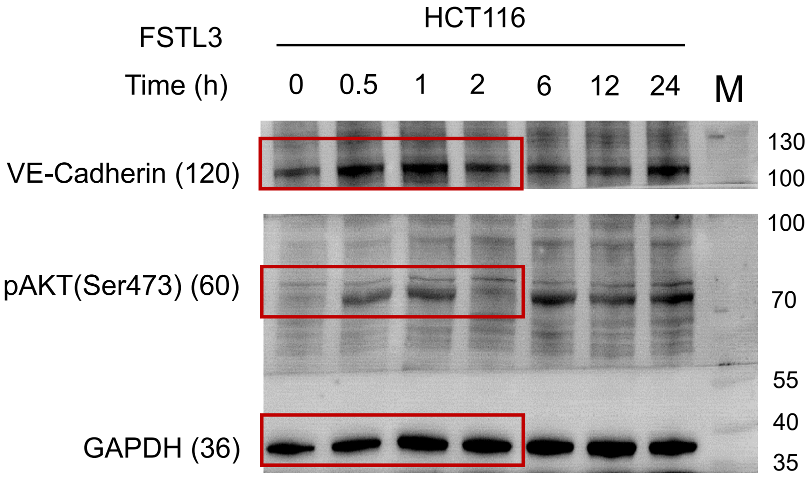

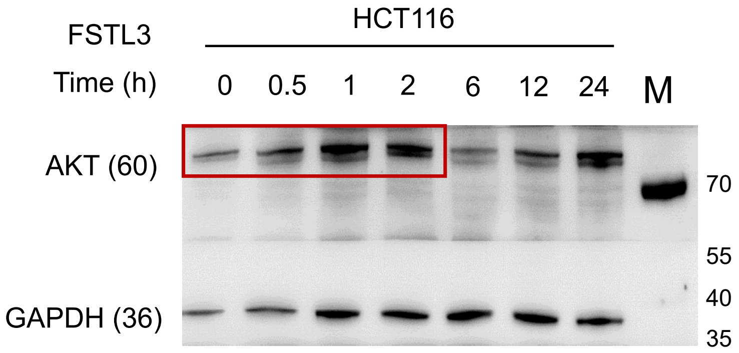

Uncropped gels for Western Blots in Figure 4H

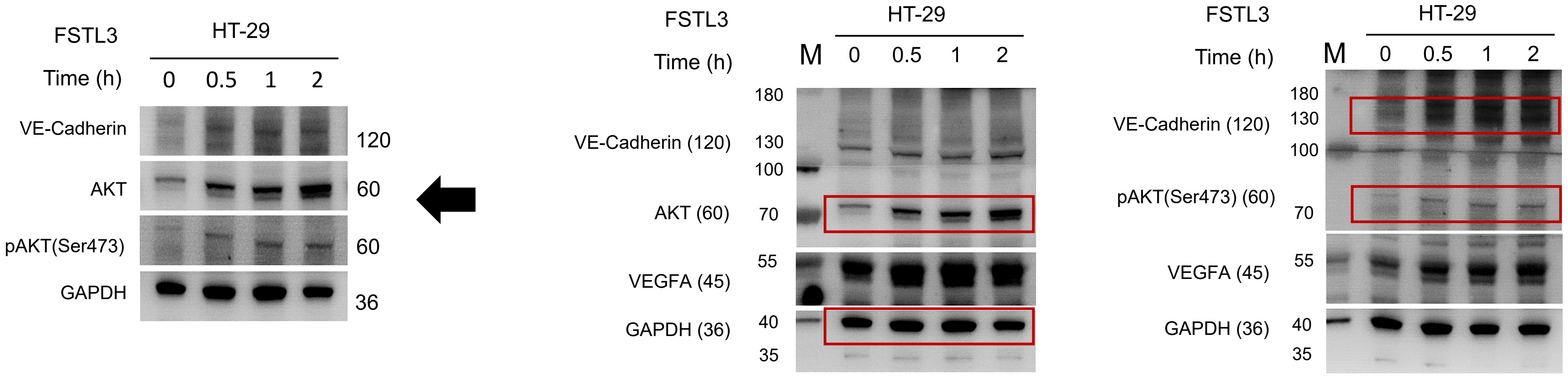

Uncropped gels for Western Blots in Figure 4K

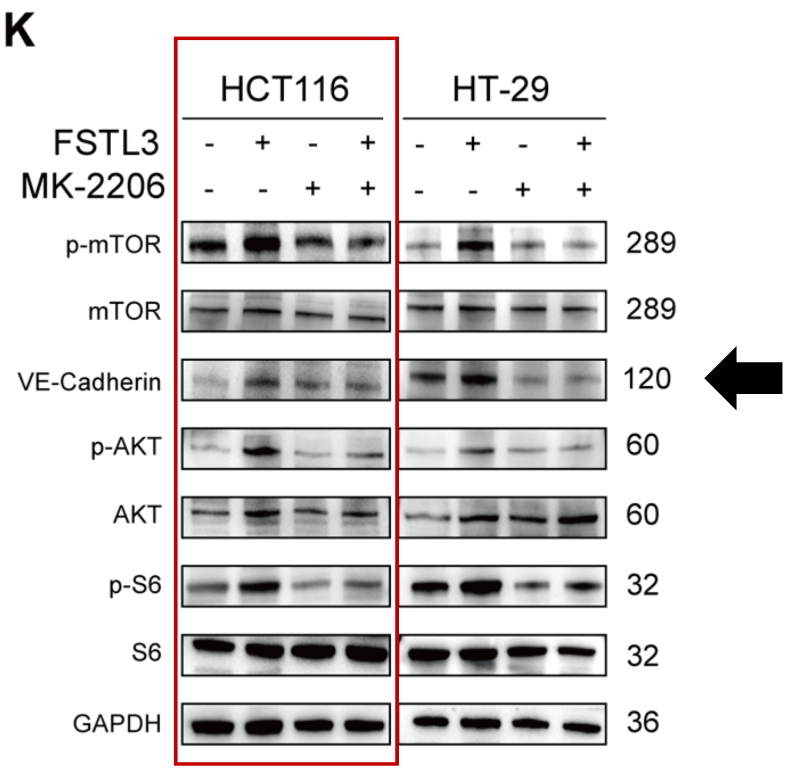

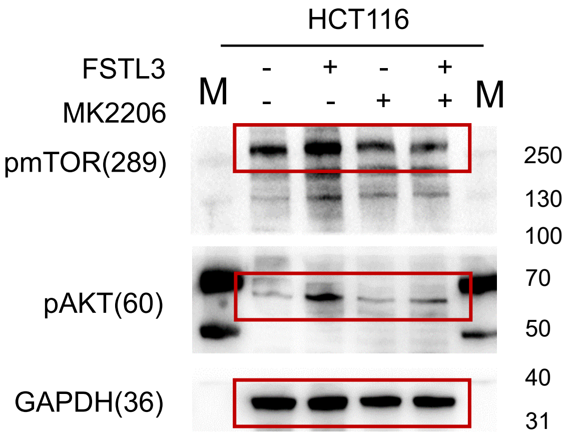

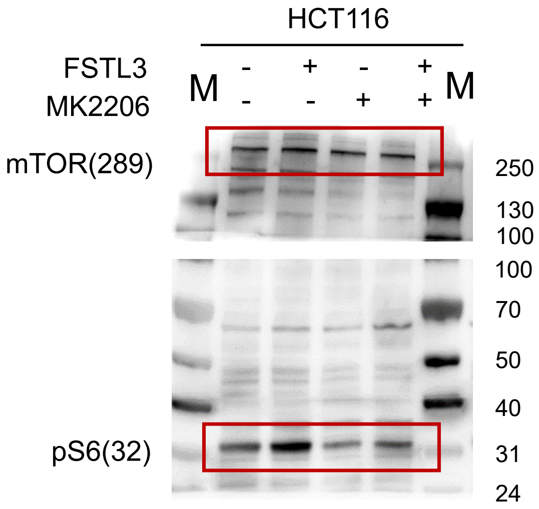

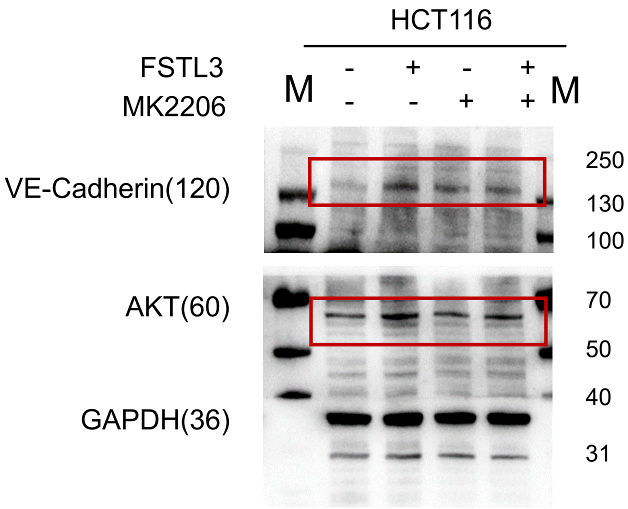

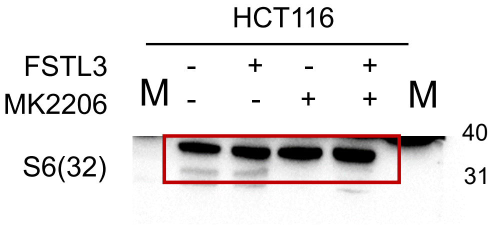

Uncropped gels for Western Blots in Figure 4K

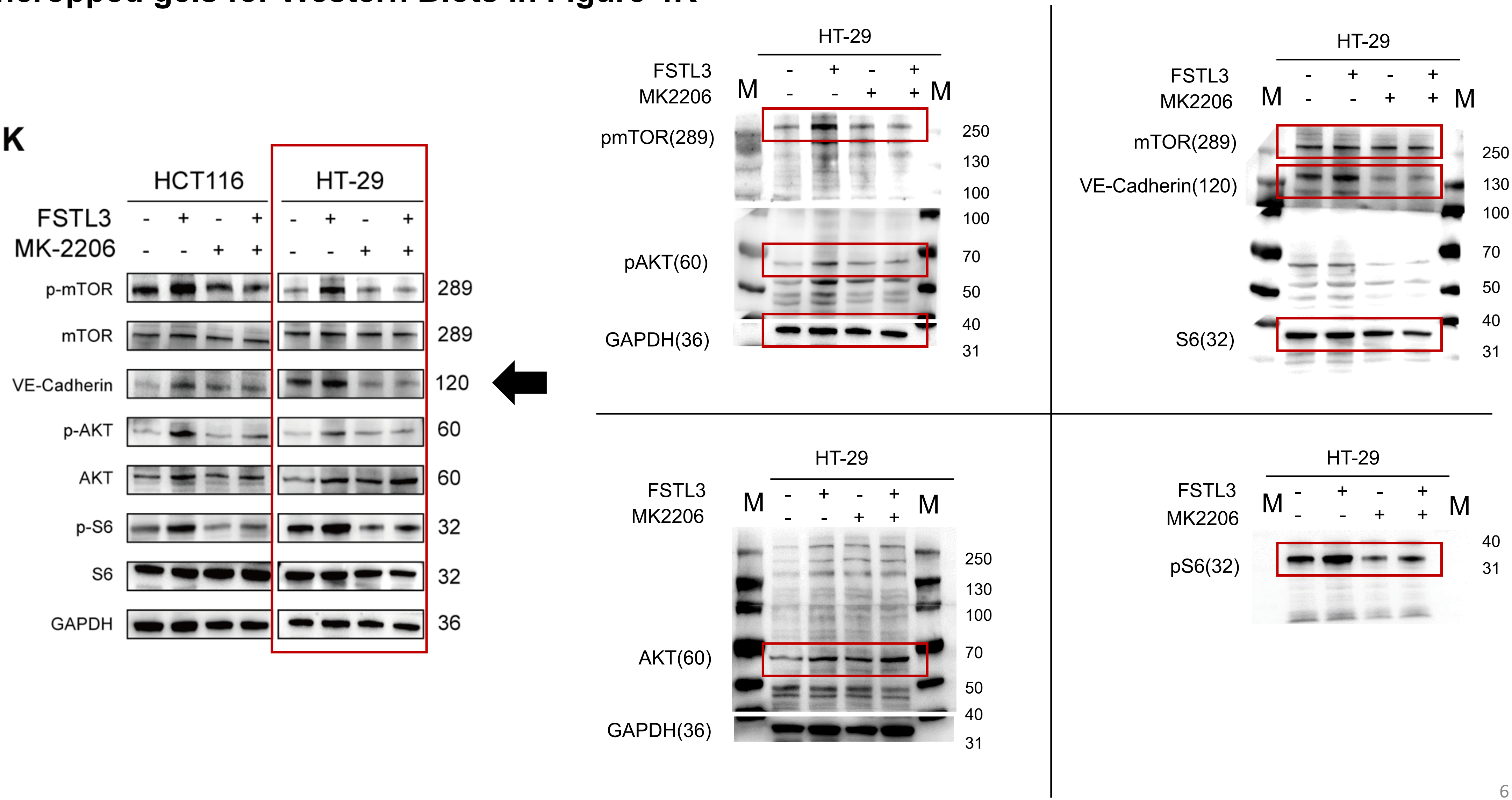

Uncropped gels for Western Blots in Figure 5D

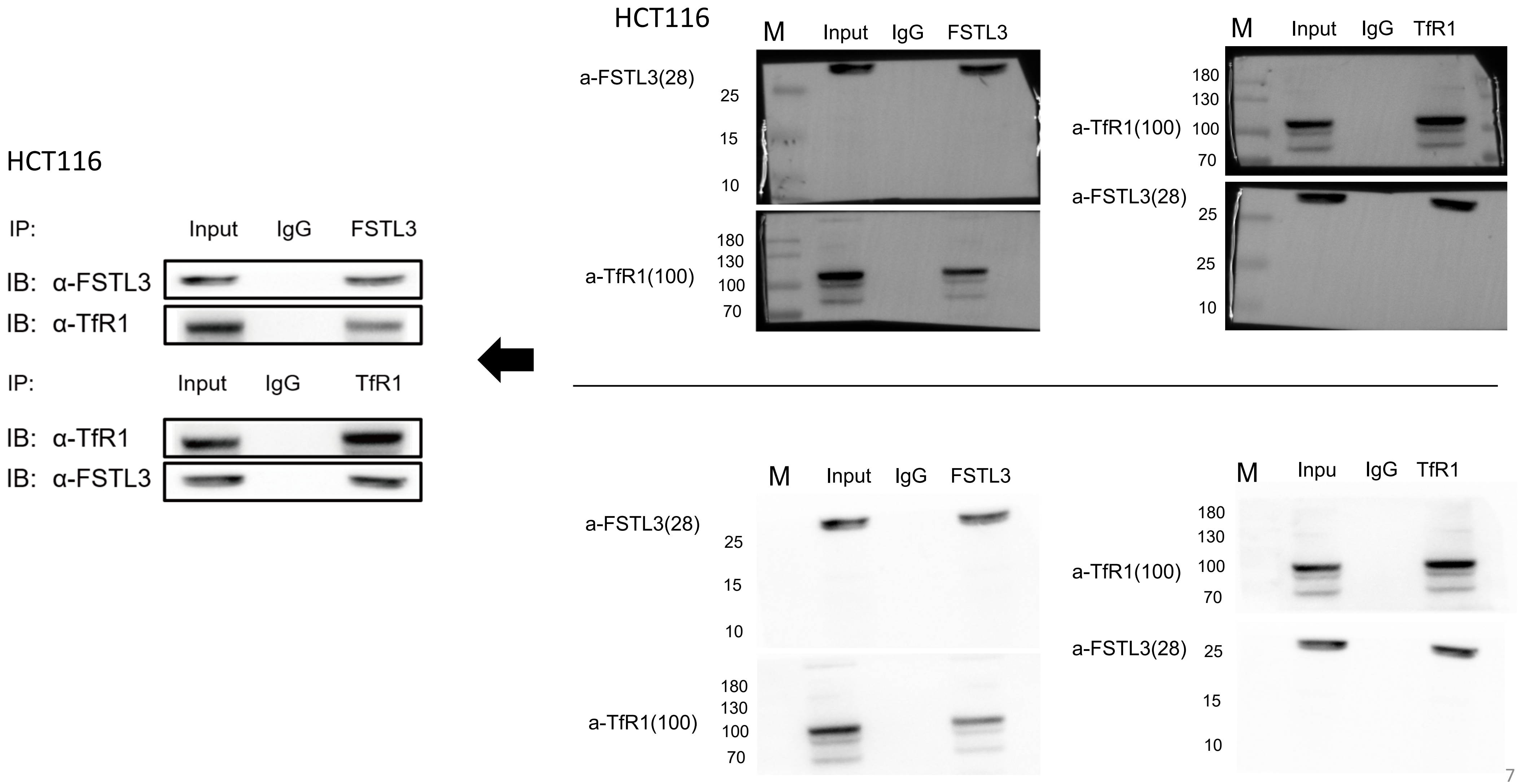

Uncropped gels for Western Blots in Figure 5I

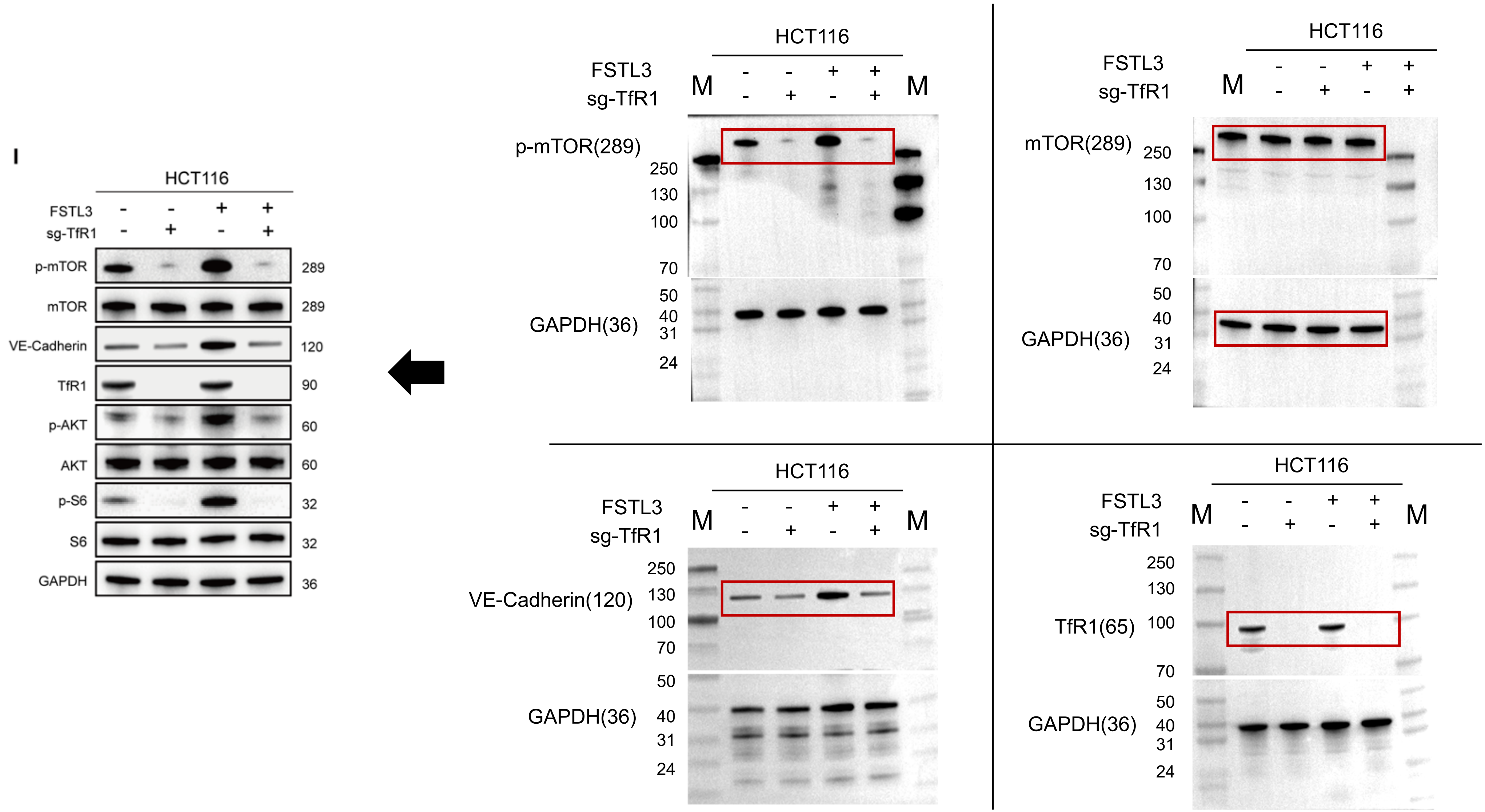

Uncropped gels for Western Blots in Figure 5I

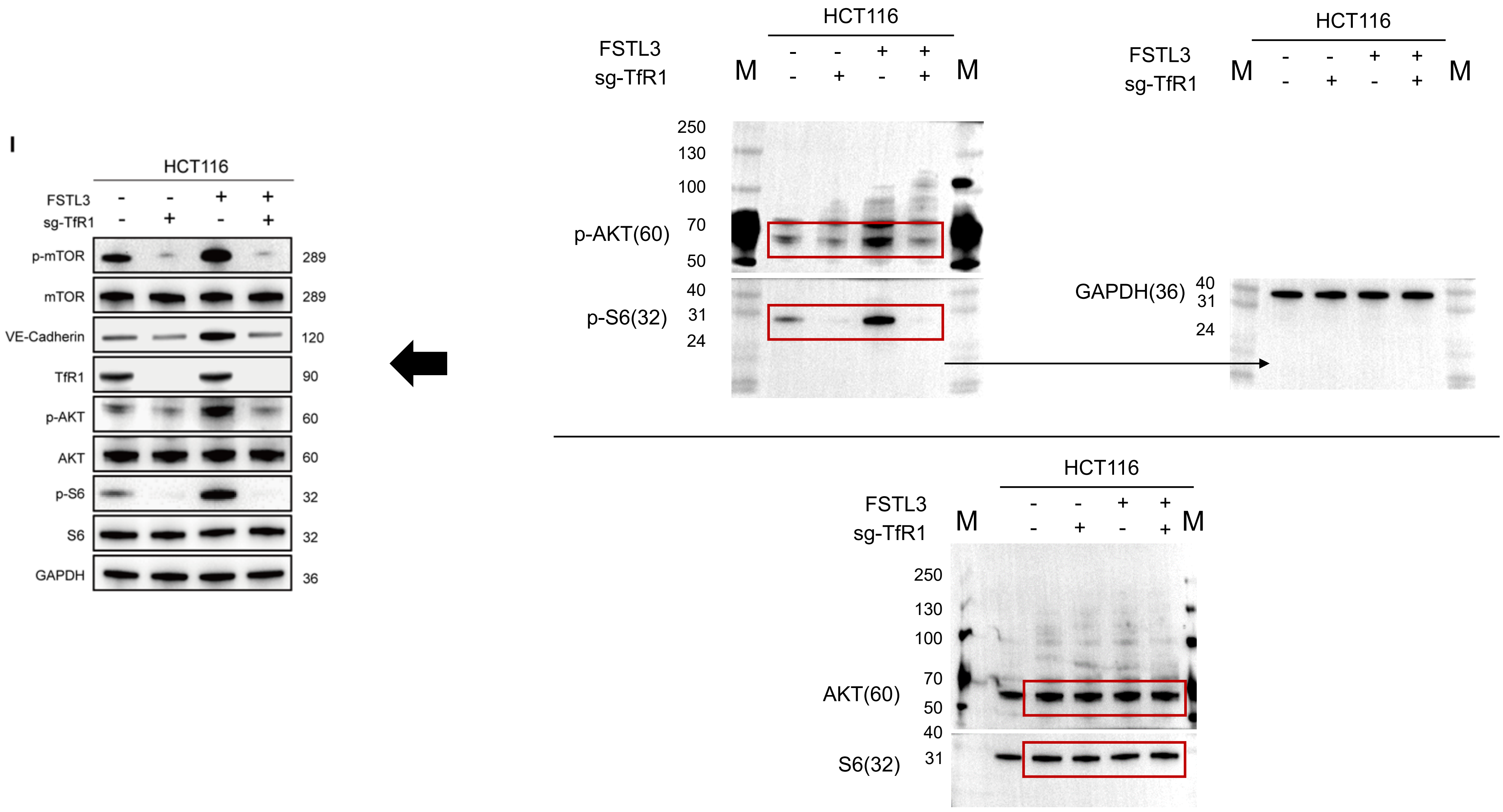

Uncropped gels for Western Blots in Figure S8D

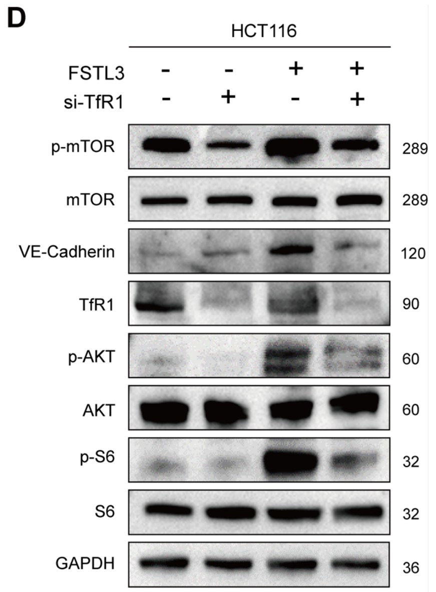

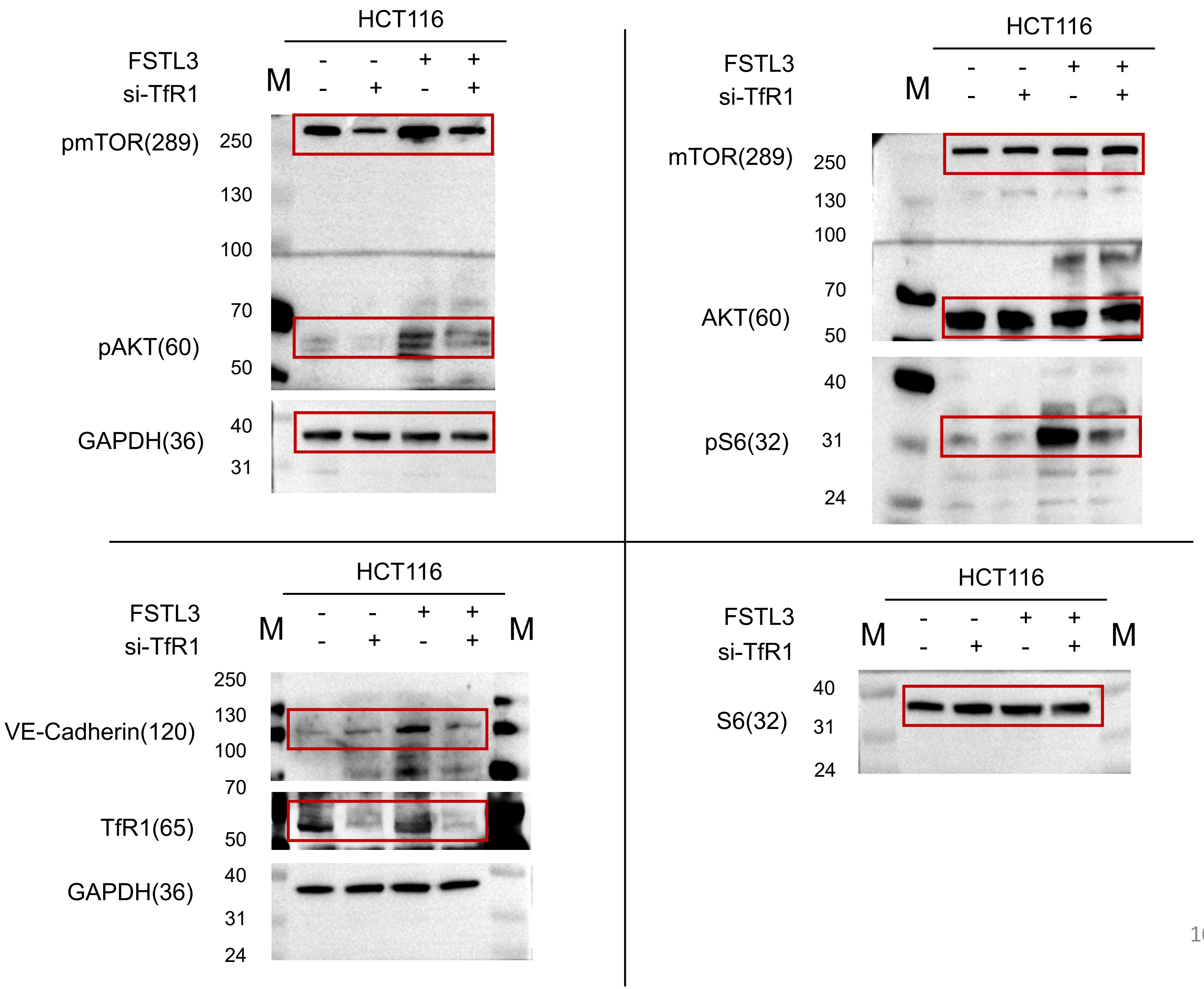

Uncropped gels for Western Blots in Figure S10D

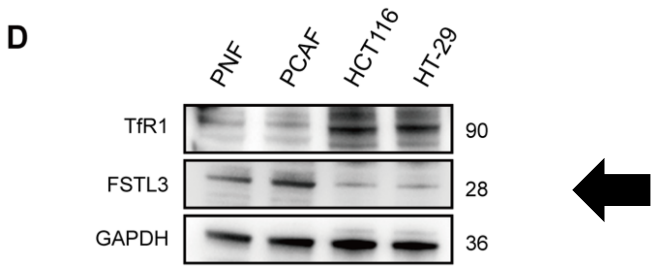

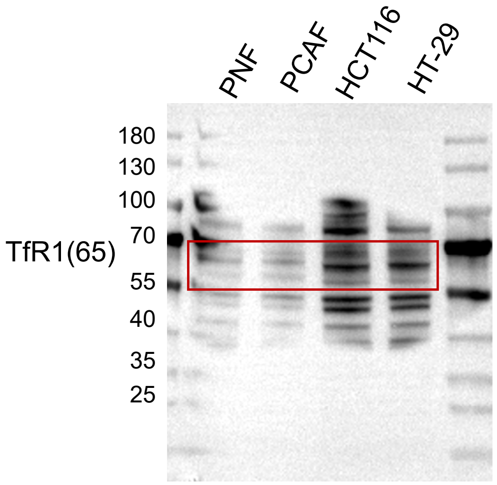

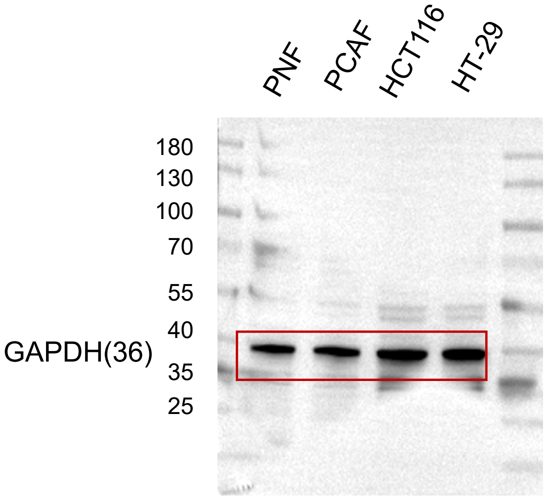

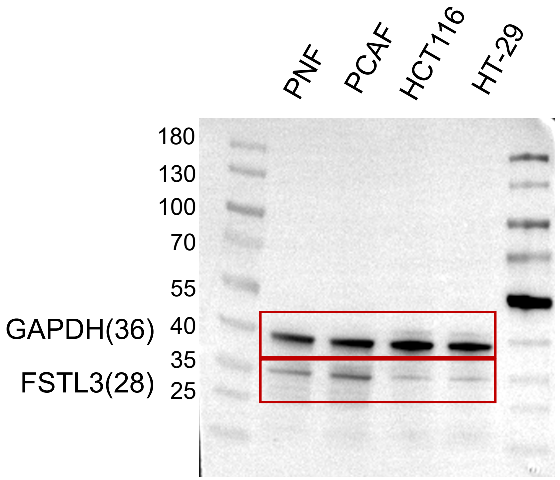

Uncropped gels for Western Blots in Figure S10F

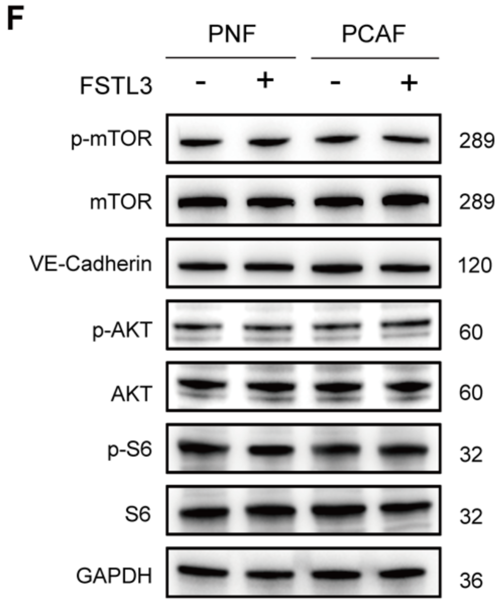

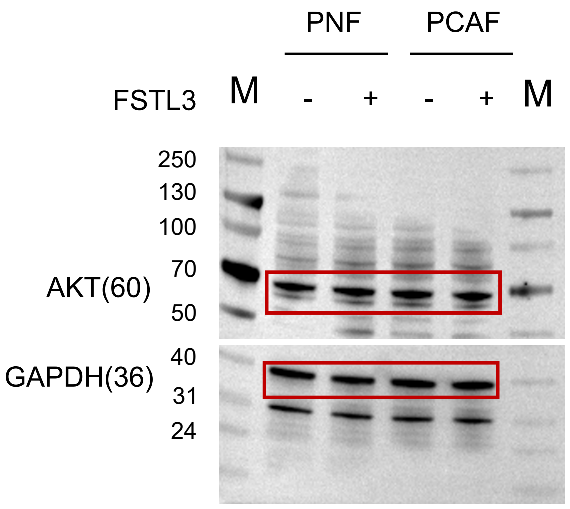

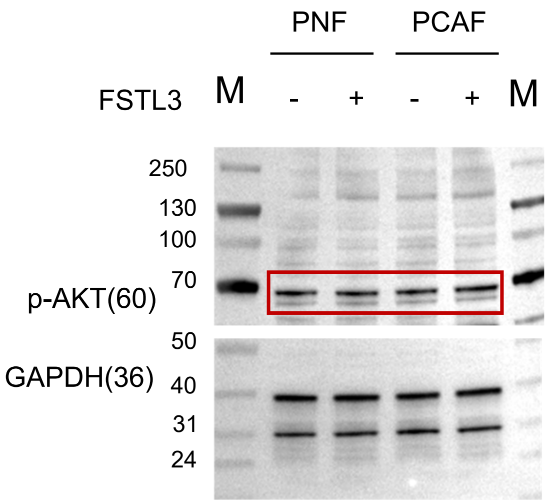

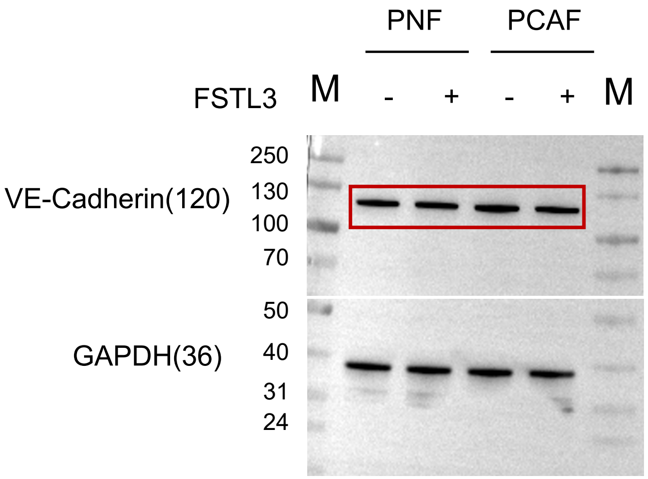

Uncropped gels for Western Blots in Figure S10F

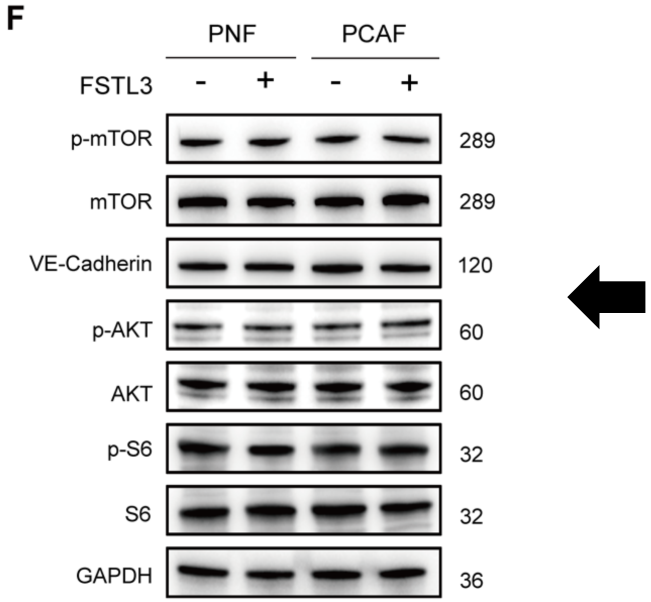

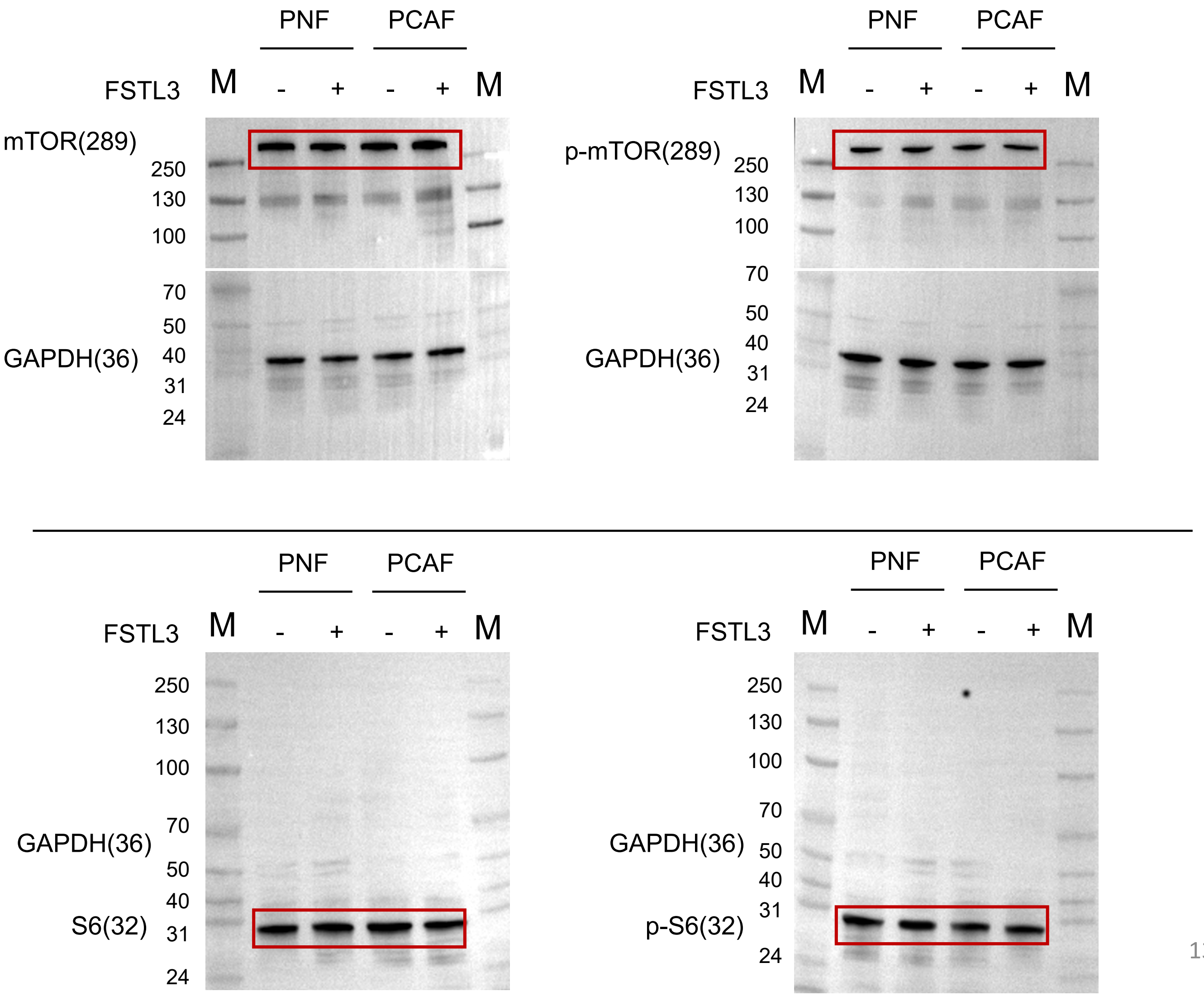

Supplement: Supplementary file 3 — Uncropped gels for Western Blots [file 41419_2025_8009_MOESM3_ESM.pdf]
